# M2 Macrophage membrane-mediated biomimetic nanoparticles carrying ADAM9 siRNA alleviate renal inflammation and fibrosis via the AKT/NF-κB pathway

**DOI:** 10.1186/s12951-026-04363-9

**Published:** 2026-05-11

**Authors:** Qingxin Li, Yue Liu, Kexin Zhou, Xiaoli Song, Kun Yue, Bing Zhou, Shengnan Fei, Xijian Wang, YouLang Zhou, Yangbo Guan, Xinzhong Huang

**Affiliations:** 1 Department of Nephrology, Affiliated Hospital of Nantong University, 20 Xisi Road, 226001 Nantong, Jiangsu China; 2 Medical School of Nantong University, Nantong 226001, China; 3 Suzhou Taihu Lake National Tourism Resort People’s Hospital, Suzhou 215000, Jiangsu China; 4 Research Center of Clinical Medicine, Affiliated Hospital of Nantong University, Nantong, 226001 China; 5 Department of Urology, Affiliated Hospital of Nantong University, Medical School of Nantong University, Nantong, China

**Keywords:** Inflammation, Renal fibrosis, Targeted drug delivery, AKT/NF-κB signaling pathways, ADAM9

## Abstract

The chronic kidney disease (CKD) situation remains severe globally. The prevention and management of CKD continue to be long-term and challenging tasks. A disintegrin and metalloprotease 9 (ADAM9) is a key factor in the progression of fibrosis and acts through multiple mechanisms, making it an important target in the study of fibrotic diseases. Thus, therapeutic strategies targeting ADAM9 hold promise for treating fibrotic disorders. This study aimed to utilize nanoparticle complexes coated with macrophage membranes (designated M2M@NP complexes) to deliver small interfering RNAs (siRNAs) targeting ADAM9 expression in the kidney. This approach was intended to exert a therapeutic effect on the progression of kidney disease. In vivo imaging confirmed that the macrophage membrane carrier exhibited excellent inflammation-targeting properties. In vitro and in vivo characterization confirmed that M2M@NPs possessed superior transfection efficiency and safety. The experimental results indicated that M2M@NP-ADAM9 siRNA complexes effectively reduced ADAM9 expression, thereby inhibiting the protein kinase B (AKT)/nuclear factor kappa-light-chain-enhancer of activated B cells (NF-κB) pathway and reducing Ras-related C3 botulinum toxin substrate 1 (RAC1) and tumor necrosis factor receptor-associated factor 6 (TRAF6) expression. These complexes also reduced macrophage infiltration and M1 polarization, leading to attenuated renal inflammation and fibrosis. Our findings suggest that the M2M@NP delivery system has broad potential for treating CKD.

**Graphical Abstract:**

**Figure:**
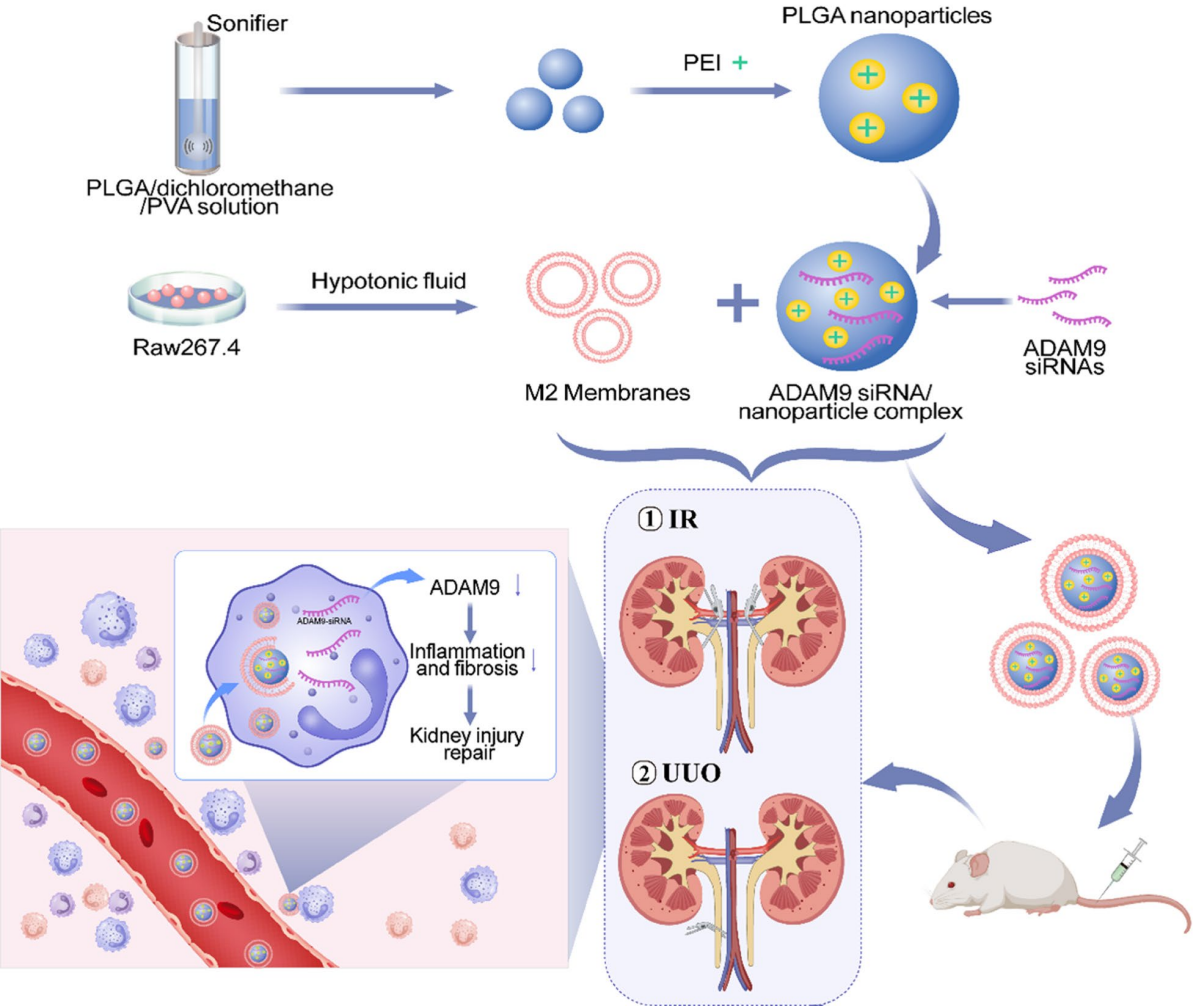
AGO@FA-lip mediated Tumor Chemoimmunotherapy and In Situ Tumor Vaccine Development (Some graphic materials were sourced from Figdraw 2.0).

## Introduction

The accelerating pace of global aging imposes considerable strain on social systems and necessitates urgent adaptations in healthcare frameworks. Notably, renal impairment associated with aging has contributed to a rising global incidence of kidney disease [1]. Acute kidney injury (AKI) is increasingly recognized as impacting not only the kidneys but also multiple organ systems throughout the body [2]. The initial insults vary; however, the pathophysiology of AKI primarily involves cell death, oxidative stress, inflammation, and microcirculatory dysfunction [3]. Although the kidneys possess remarkable reparative capabilities following acute injury, severe or persistent damage can lead to incomplete or maladaptive repair, ultimately resulting in chronic kidney disease (CKD). The repair process in damaged kidneys is highly complex, with inflammation playing a crucial role [4]. Following AKI, rapid and robust activation of multiple signaling pathways occurs in intrinsic renal cells, which subsequently results in the secretion of multiple inflammation-related cytokines and mediators. Key receptor families, such as Toll-like receptors (TLRs), receptor tyrosine kinases, G protein-coupled receptors, and peroxisome proliferator-activated receptors, are activated upon sensing kidney injury. This triggers a series of downstream signaling events, including the activation of NF-κB pathways, which are involved in regulating inflammation and cell survival; phosphatidylinositol 3-kinase (PI3K)/Akt pathways, which promote cell survival and proliferation; and mitogen-activated protein kinase family members, which impact cell cycle progression, differentiation, and inflammatory responses [5].

AKI is a critical clinical emergency with a high mortality rate, exceeding 50% in intensive care unit settings [6, 7]. Severe AKI or AKI that does not effectively resolve can result in irreversible structural and functional damage to the kidneys. When renal tubular cells undergo significant damage during AKI, their regenerative and reparative capacities may be compromised, leading to tubular degeneration. Prolonged inflammatory stimulation further accelerates tubulointerstitial fibrosis, culminating in renal fibrosis [8–11]. ADAM9 can cleave membrane-bound pro-inflammatory cytokine precursors (e.g., TNF-α and IL-6), converting them into their active forms and releasing them into the extracellular space. This process continuously activates local renal immune cells (e.g., macrophages), leading to inflammatory cell infiltration. High ADAM9 expression sustains a state of chronic renal inflammation by activating the TLR signaling pathway, the IL-17 signaling pathway, and the NOD-like receptor signaling pathway, thereby accelerating the chronic progression of renal injury. Fibrosis represents the core pathological change during the progression from AKI to CKD. ADAM9 can activate the AKT/NF-κB signaling pathway, causing renal tubular epithelial cells to lose their epithelial phenotype and transdifferentiate into a fibroblast-like phenotype [12, 13]. These cells subsequently secrete large amounts of extracellular matrix (ECM) components, such as Col-1 and α-SMA. Excessive deposition of these ECM components in the renal interstitium ultimately leads to tubulointerstitial fibrosis, disrupting the normal structural architecture of the kidney [14]. Clinical and experimental evidence has indicated a strong interrelationship between AKI and CKD; AKI can progress or transition to CKD, while CKD sensitizes the kidneys to AKI and hinders renal repair following AKI [10, 15]. Therefore, controlling inflammation is essential for treating renal injury and serves as a critical strategy for preventing fibrosis.

A disintegrin and metalloprotease 9 (ADAM9) is a membrane-anchored protein that plays a critical role in various physiological functions. Its disintegrin domain is used for cell adhesion, and its metalloproteinase domain is involved in shedding extracellular domains from multiple cell surface proteins. ADAM9 is involved in developmental processes, inflammatory responses, and degenerative diseases [16]. It has been shown to stimulate inflammatory processes involving polymorphonuclear leukocytes, macrophages, and epithelial cells under certain inflammatory conditions. Additionally, ADAM9 can induce apoptosis, as observed in acute lung injury and chronic obstructive pulmonary disease [17–20]. In the epidermis, particularly in keratinocytes, ADAM9 was reported to regulate wound healing by reducing keratinocyte migration and slowing the healing process by increasing collagen XVII shedding and matrix metalloproteinase-9 (MMP-9) secretion [21]. Therefore, inhibiting ADAM9 may alleviate inflammation during AKI and attenuate renal fibrosis.

RNA interference (RNAi) is a natural defense mechanism that protects against foreign nucleic acids and regulates gene expression [22]. Small interfering RNAs (siRNAs) are RNAi mediators in mammalian cells [23]. These siRNAs serve as powerful tools for gene function research and potential therapeutic applications by suppressing specific gene expression. The first siRNA drugs were approved by the U.S. Food and Drug Administration and the European Medicines Agency between 2018 and 2022, 20 years after the discovery of RNAi [24]. Optimal siRNAs should not activate the innate immune system, but should efficiently and specifically cleave their targets while minimizing off-target effects (i.e., influence on non-target genes) and other toxicities. They should also possess long half-lives and slow degradation rates within the body and target cells. Selecting suitable silencing targets is crucial for treating specific diseases. To date, most therapeutic siRNAs have targeted protein-coding messenger RNAs [25]. Poly(lactic‒-co-glycolic acid) (PLGA) nanoparticles exhibit significant biocompatibility, non-toxicity, film-forming properties, and biodegradability. They have demonstrated benefits in multiple fields, including medicine and engineering [26].

**Fig. 1:**
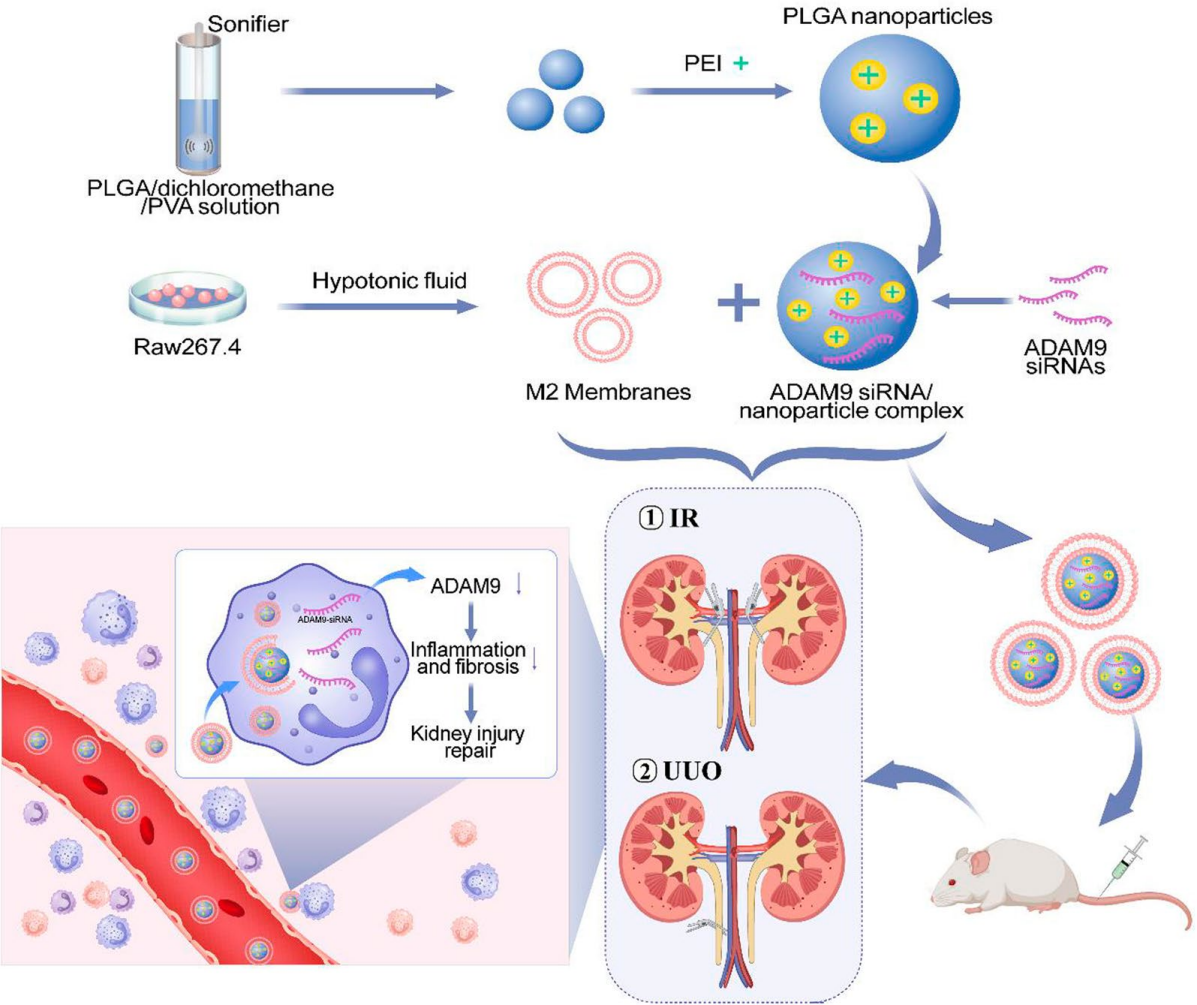
A schematic diagram illustrating the procedure for preparing the M2M@NP-ADAM9 siRNA delivery system and the therapeutic mechanism by which this system alleviates AKI and CKD by silencing ADAM9

Recently, biomimetic nanocarrier delivery systems have gained increasing attention [27, 28]. These systems transfer bioactive cell membranes onto the surface of nanoparticles and maintain biocompatibility while combining the targeting efficacy of membranes with the unique physical properties of conventional nanoparticles [29, 30]. Macrophages are crucial immune cells that play key roles in inflammatory responses, tissue repair, and homeostasis maintenance. Macrophages originating from monocytes in the blood can polarize into different functional states depending on their microenvironment and the signals they receive. The most common types are M1 and M2 macrophages. M1 macrophages phagocytose pathogens and promote inflammation, whereas M2 macrophages are involved in tissue repair, wound healing, and angiogenesis [31, 32]. Given these characteristics, the M2 macrophage membrane has the potential to target inflamed areas. In our study, we induced primary macrophages to the M2 phenotype and successfully extracted cell membranes. The extracted M2 macrophage membranes (M2Ms) were combined with PEI/PLGA nanoparticles to construct the M2M@NP-ADAM9 siRNA delivery system. Although the positively charged PEI-modified nanoparticles can electrostatically interact with the negatively charged macrophage cell membranes, the extrusion method employed in this study (membrane extrusion through 100–200 nm pores) is able to recapitulate the native physiological state of cell membranes, thereby forcing the membranes to bind to the nanoparticles in a right-side-out orientation. This fabrication strategy has been validated by numerous studies to stably preserve the functional orientation of the membrane and to prevent charge-driven orientation disorder. Meanwhile, zeta potential measurements of the membrane-coated nanoparticles showed a negative surface charge. This finding is consistent with the intrinsic surface properties of native macrophage membranes andprovides indirect evidence for the stability of the right-side-out configuration [33]. We evaluated its targeting specificity and therapeutic efficacy in mouse models subjected to bilateral renal ischemia‒reperfusion (I/R) and unilateral ureteral obstruction (UUO). We hypothesize that this system can target inflammatory sites following injury and release siRNA encapsulated in PEI/PLGA nanoparticles to exert an effect. Figure 1 outlines the rationale and methodology behind our research. Our delivery system offers an effective biological method for mitigating AKI via CKD. M2M@NPADAM9 siRNA.

## Materials and methods

### Materials and animals

Three small interfering RNAs (siRNAs) with the following sequences were obtained from Suzhou GenePharma Co., Ltd. (Suzhou, China):

**Table:** 

Adam9-mus-243	GGAACAGACUGUCCAUCUUTT	AAGAUGGACAGUCUGUUCCTT
Adam9-mus-1191	GCAUAACCUUGGAAUGAAUTT	AUUCAUUCCAAGGUUAUGCTT
Adam9-mus-2698	GCUCCUGCACCUCCUUUAUTT	AUAAAGGAGGUGCAGGAGCTT

Creatinine and urea nitrogen were measured using commercial assay kits (JianCheng, C011—2—1; Nanjing, China). The antibodies used in this study included ADAM9, nuclear factor (NF)-κB p65, phosphorylated Akt (P-Akt), fibronectin, alpha-smooth muscle actin (α-SMA), collagen I (Col-I), interleukin-6 (IL-6), tumor necrosis factor-α(TNF-α), cluster of differentiation 206 (CD206), cluster of differentiation 14 (CD14), Ras-related C3 botulinum toxin substrate 1 (Rac1), tumor necrosis factor receptor–associated factor 6 (TRAF6), and glyceraldehyde-3-phosphate dehydrogenase (GAPDH). All antibodies were obtained from Proteintech Group, Inc. (USA). C57BL/6 mice (male, 8 weeks old, 19–23 g) and specific pathogen-free Sprague–Dawley rats (SD; male, 12 weeks old, 210–260 g) were obtained from the Nantong University Experimental Animal Center (China). All animals were housed under controlled conditions, with a relative humidity of 50–60%, a temperature of 20–25 °C, and a 12-h light/dark cycle. Animals had free access to food and water.

### Data collection and processing

Gene transcription profiles and the associated clinical data of CKD patients, including eight normal kidney samples and 53 CKD samples, were obtained from the Gene Expression Omnibus (GEO) database. Samples lacking critical clinical information were excluded from further analysis. R software (available at https://www.r-project.org/) was used to analyze the genetic profiles and corresponding clinical data of the remaining tissue samples. Within the GEO database, the Limma, sva, ggplot2, pheatmap, dplyr, and ggexceller packages were utilized to identify the differentially expressed genes (DEGs) between the normal kidney tissue samples and the CKD samples. Genes with an absolute log_2_ fold change (|log₂FC|) > 1 and a *P* value < 0.05 were considered statistically significant. The expression of these DEGs was further validated using the GSE62792 dataset, which consists of 12 normal kidney tissue samples and six CKD samples.

### HK-2 cell culture

HK-2 cells were cultured in Dulbecco ‘s Modified Eagle ‘s Medium/Nutrient Mixture F-12 (DMEM/F-12) supplemented with 10% fetal bovine serum and 1% penicillin–streptomycin. HK-2 cells were incubated at 37 °C with 5% CO_2_ in a cell culture incubator at constant temperature and humidity. HK-2 cells were seeded onto 6-well plates and treated with hydrogen peroxide (H_2_O_2_) or transforming growth factor-beta (TGF-β) to establish in vitro models of inflammation and fibrosis, respectively.

### siRNA selection

HK-2 cells were seeded into 6-well plates at 70–80% confluence and allowed to grow overnight. Three siRNAs were chosen, and 1 OD of siRNA was dissolved in 125 μL of diethyl pyrocarbonate (DEPC)-treated water to prepare a 20 µM stock solution. siRNA (8 μL/well) was mixed with 8 μL/well of nanoparticles and incubated at 25 °C for 25 min to form PEI/PLGA-siRNA complexes. The resulting mixtures were then added to the wells and gently agitated followed by incubation for 24–48 h. Cells were divided into four treatment groups: normal control (NC), siRNA2698, siRNA243, and siRNA1191. After treatment, proteins were extracted for subsequent Western blot analysis.

### Transcriptome sequencing

In this study, total RNA containing poly(A) tails was enriched using oligo(dT) magnetic beads. The RNA was fragmented into approximately 300 bp segments via ion shearing, and fragments of approximately 300 bp were selected. First-strand cDNA was synthesized using random hexamers and reverse transcriptase, followed by synthesis of the second-strand cDNA. A sequencing library was then constructed and amplified by polymerase chain reaction (PCR), with 450 bp fragments selected for the library. The quality of the library, as well as its total and effective concentrations, was determined. Libraries with different index sequences were then mixed. The pooled libraries were diluted to a concentration of 2 nM and then denatured to generate single-stranded libraries. After the samples underwent RNA extraction, purification, and library preparation, the samples were sequenced on the Illumina platform using paired-end sequencing.

### Raw data processing, filtering, and quality assessment

After sequencing, image files were generated and converted by the sequencing platform ‘s proprietary software into FASTQ format raw data (raw data). Raw data statistics, including sample names, Q30 scores, the proportion of undetermined bases, and the Q20 (%) and Q30 (%) values, were compiled for each sample. The raw sequencing data contained adapter sequences and low-quality reads, which could interfere with downstream analyses. Therefore, a series of filtering steps was applied to the raw data. The primary criteria for data filtering included using Fastp to remove sequences with adapter contamination at the 3 ‘ ends and to exclude reads with an average quality score below Q20.

### Functional enrichment analysis of differential expression gene

TopGO was utilized for Gene Ontology (GO) enrichment analysis to elucidate the functional annotation of genes with altered expression levels. By employing GO term annotations, the analysis enumerates the differential genes and their frequencies within each term. Significance levels are assessed using the hypergeometric distribution method, where a threshold *P* value of < 0.05 indicates significant enrichment. This methodology enables the identification of GO terms that are overrepresented among the differentially expressed genes, providing insight into their primary biological functions. The enrichment significance is quantified using the rich factor, the false discovery rate (FDR), and the number of genes enriched within each GO term. The rich factor is defined as the proportion of differentially expressed genes enriched in a specific GO term in the total number of genes associated with that term. Higher rich factors reflect more significant enrichment, and an FDR value approaching 0 indicates a higher level of statistical significance in the enrichment. The analysis also incorporates Kyoto Encyclopedia of Genes and Genomes (KEGG) pathway enrichment results, highlighting the top 20 GO terms and KEGG pathways characterized by the lowest FDR values.

### Primary macrophage cultivation

Ten-week-old SD rats were subjected to an overnight fast. Euthanasia was performed via intraperitoneal injection of pentobarbital (100 mg/kg) to ensure a rapid and humane endpoint. After surface sterilization, the rats were transferred to a sterile workbench. The abdominal muscles were exposed and disinfected, and the peritoneal cavity was rinsed with prewarmed PBS. The abdomen was massaged for 3 min and allowed to rest for 5 min. A small incision was then made in the abdominal muscle using sterile scissors. The peritoneal fluid and medium were collected and centrifuged at 1000 rpm for 5 min to pellet the cells. The cells were resuspended in RPMI 1640 medium and inoculated into culture dishes. Recombinant human IL-4 (20 ng/mL; Proteintech) was added to induce primary macrophages to differentiate into M2 macrophages within 24 h.

### Preparation of M2 macrophage membranes

First, the primary M2 macrophages were washed with PBS after removing the culture medium. The samples were then resuspended in precooled homogenization buffer containing Tris–HCl, KCl, sucrose, MgCl2, and protease/phosphatase inhibitors. The cells were lysed using a JY 92-IIN homogenizer at 75 W. The resulting lysate was initially centrifuged at low speed (3,000 × g, 4 °C) for 10 min, followed by high-speed centrifugation (10,000 × g, 4 °C) for 30 min. The collected supernatant was mixed with VEX exosome extraction reagent (one-third volume) and maintained at 4 °C for 24 h. Next, the sample was centrifuged at 10,000 × g at 4 °C for 1 h, and the pellet, enriched with M2 macrophage membranes, was collected. A BCA assay kit was used to analyze the concentration of membrane proteins in the collected fraction, and the M2 macrophage membrane product containing approximately 1 mg of membrane protein was kept in water at 4 °C.

### Preparation of FITC-M2Ms

For fluorescent labeling, fluorescein isothiocyanate (FITC) was added to the membrane suspension at a final concentration of 20 μg/mL, followed by incubation at 4 °C for 1 h with gentle shaking in the dark. Unbound free FITC was removed by three cycles of centrifugation at 14,000 g for 20 min at 4 °C and resuspension in fresh PBS. Finally, FITC-labeled M2-type macrophage membranes (FITC-M2Ms) were collected and stored at − 80 °C until further use.

### Preparation of PLGA nanoparticles

In a clean glass container, 100 mg of PLGA powder was dissolved in 1 mL of dichloromethane to prepare a 10% (w/v) PLGA solution. Two PVA aqueous solutions were prepared at concentrations of 7% and 1%. The two solutions were mixed and sonicated for 1 min to form a primary emulsion. This initial emulsion was slowly dripped into 50 mL of 1% PVA aqueous solution while undergoing further sonication. The mixture was continuously stirred at 25 °C for 12 h, allowing the dichloromethane to gradually evaporate, thus enabling PLGA to coalesce into nanoparticles. The mixture was washed, centrifuged, resuspended, and maintained at 4 °C.

### Preparation of PEI/PLGA-siRNA complexes

To load siRNA onto nanoparticles, the nanoparticles were first modified with PEI to impart a positive charge, thereby facilitating the attraction of negatively charged siRNA. Briefly, 100 μL of PLGA nanoparticle solution (10 μg/μL) was mixed with 200 μg of PEI (100 μg/μL) in deionized water. The mixture was then added to a solution with a nitrogen-to-phosphate (N/P) ratio of 6:1 and gently stirred for 20 min to form stable PEI/PLGA-siRNA complexes.

### Preparation and characterization of M2M@NP complexes

Equal amounts of M2M and PEI-functionalized PLGA nanoparticles were mixed and repeatedly filtered through polycarbonate membranes with pore sizes of 0.45 μm and 0.2 μm. The M2M, PEI/PLGA nanoparticles, and the M2M@PLGA complex structures were observed by scanning electron microscopy (SEM). The particle sizes were measured using a Mastersizer 3000 laser particle size analyzer, and the zeta potentials were measured using a Zetasizer Nano ZS.

### Colocalization studies

Rhodamine B-labeled PEI/PLGA and fluorescein isothiocyanate (FITC)-labeled M2Ms were prepared in M2M@PLGA complexes using ultrasonic treatment. The samples were then centrifuged at 1000 × g for 10 min to separate the desired complexes. Confocal laser scanning microscopy imaging of the M2M@PLGA complexes was performed via a Leica TCS SP8 STED 3X microscope (Leica Microsystems, Mannheim, Germany).

### Flow cytometry

HK-2 cells were grown in DMEM supplemented with 10% FBS for 48 h and then rinsed three times with PBS. A fluorescence microscope (Leica DMR 3000) with a FITC channel (excitation 488 nm, emission 518 nm) was used for the preliminary observation of successful transfection and the expression of fluorescently labeled proteins in the cells. The cells were subsequently harvested and suspended in PBS for quantitative analysis of transfection efficiency. Finally, the transfection efficiency was precisely calculated by flow cytometry using a FACSCalibur instrument to determine the proportion of successfully transfected and fluorescently expressing cells among the total cell population.

### Animal models

The method for establishing a mouse I/R model is well practiced [34]. In brief, the mice were subjected to general anesthesia, followed by a skin incision. The renal pedicles were exposed through blunt dissection and clamped for 45 min. During the clamping period, the mice were placed in a warm environment. After surgery, the mice were returned to their cages. The sham surgery group underwent only the incision and suturing procedures.

UUO was established according to an earlier report [35]. The mice were subjected to general anesthesia, and the surgical site was disinfected. A small longitudinal cut was made in the flank region to expose the ureter, which was then completely ligated. Finally, the incision was closed with sutures. In the sham operation group, all steps were performed except for ligating the ureter.

### Animal experimental design

The I/R model mice were randomly allocated to seven groups (n = 6/group): the sham group (sham operation group, in which the mice received a sham laparotomy); the I/R group; the I/R-PLGA group (treated with a tail vein injection of 0.1 mL M2M@NPs immediately after surgery-induced I/R); the I/R-NC group (treated with a tail vein injection of 0.1 mL M2M@NP-20 µg NC siRNA after surgery-induced I/R); the 5 µg ADAM9 siRNA group (treated with a tail vein injection of 0.1 mL M2M@NP-5 µg ADAM9 siRNA after surgery-induced I/R); the 10 µg ADAM9 siRNA group (treated with a tail vein injection of 0.1 mL M2M@NP-10 µg ADAM9 siRNA after surgery-induced I/R); and the 20 µg ADAM9 siRNA group (treated with a tail vein injection of 0.1 mL M2M@NP-20 µg ADAM9 siRNA after surgery-induced I/R). Drug injections of 0.1 mL were administered immediately after suturing in the I/R model mice. Kidneys and blood were collected 24 h post-surgery. The bilateral upper and lower portions of both kidneys were carefully dissected from each group and promptly stored in a -80 °C freezer. After the kidney tissue was excised, a portion of the mid-kidney was immediately placed in electron microscopy fixative and sent for electron microscopy examination. The remaining tissue was fixed in 4% paraformaldehyde for preservation and analysis.

UUO mice were randomly allocated to seven groups (n = 6/group): the sham group (sham operation group); UUO group; UUO-PLGA group (received weekly tail vein injections of 0.1 mL M2M@NP post-surgery-induced UUO); UUO-NC group (received weekly tail vein injections of 0.1 mL M2M@NP-20 µg NC siRNA post-surgery-induced UUO); 5 µg ADAM9 siRNA group (received weekly tail vein injections of 0.1 mL M2M@NP-5 µg ADAM9 siRNA post-surgery-induced UUO); 10 µg ADAM9 siRNA group (received weekly tail vein injections of 0.1 mL M2M@NP-10 µg ADAM9 siRNA post-surgery-induced UUO); and 20 µg ADAM9 siRNA group (received weekly tail vein injections of 0.1 mL M2M@NP-20 µg ADAM9 siRNA post-surgery-induced UUO). The mice from each group were randomly euthanized on day 14, and samples were taken. Residual urine pressure compressing the renal pelvis was evident in obstructed kidneys, confirming the effective generation of the UUO model.

### Assessment of* In Vivo* targeting to damaged regions

We established I/R and UUO animal models to evaluate the targetingability of M2M@NP complexes to damaged areas. Immediately post-surgery, the mice received a 200 µL injection of M2M@NPDir via the tail vein. At 24 h, we performed in vivo imaging using the Tanon ABL X5 (Tanon Science & Technology Co., Ltd., Shanghai, China). An overdose of an anesthetic agent was used to euthanize the mice, and the hearts, lungs, spleens, livers, and kidneys were removed and imaged.

### Protein blotting analysis

HK-2 cells or renal tissue samples were processed using RIPA lysis buffer containing protease inhibitors. The lysates were heated to denature proteins. Following sodium dodecyl sulfate‒polyacrylamide gel electrophoresis, the proteins were transferred to polyvinylidene fluoride membranes and blocked with rapid blocking solution for 20 min. After blocking, the samples were incubated with diluted primary antibodies at 4 °C for 16 h. The following day, the sections were incubated with horseradish peroxidase-conjugated secondary antibodies at 25 °C for 2 h. Finally, the reactions were visualized using a chemiluminescent reagent (WBKLS0100; Millipore).

### Masson ‘s trichrome staining and H&E staining

Kidney tissues from mice in the different groups were fixed in 4% paraformaldehyde at 25 °C for 24 h. The kidney tissue samples were subsequently dehydrated, embedded in paraffin blocks, sectioned, and rehydrated. Standard protocols (Solarbio, Beijing, China) were followed for Masson ‘s trichrome and hematoxylin and eosin (H&E) staining. Images were developed from the stained preparations and analyzed for inflammation, adhesion formation, and collagen fibers. Multiple organs from the mice in each group were subjected to H&E staining to assess the M2M@NP-siRNA delivery system.

### TUNEL assay

TUNEL staining is a method used to detect and quantify DNA fragmentation during cell apoptosis. Renal tissue sections were treated with proteases to increase cell membrane permeability and then treated with H_2_O_2_. The samples were placed in a reaction mixture containing terminal deoxynucleotidyl transferase and biotin-labeled deoxyuridine triphosphate and incubated at room temperature for approximately 1 h. The streptavidin-enzyme complex was then added, followed by counterstaining with hematoxylin before mounting for observation.

### Immunofluorescence

The tissue sections were treated with 0.3% Triton X-100-containing blocking solution for 90 min and incubated overnight at 4 °C with primary antibodies against fibronectin, α-SMA, Col-1, TNF-α, IL-6, and ADAM9. The samples were incubated with diluted fluorescently labeled secondary antibodies at 25 °C in the dark for 2 h. The sections were gently washed with PBS buffer, stained with 4 ‘,6-diamidino-2-phenylindole (DAPI) to label the cell nuclei, and observed via fluorescence microscopy. The results were recorded.

### Statistical analysis

The data are presented as the means ± standard deviations. Logarithmic transformations or nonparametric analyses were used for datasets involving three or more groups if the variances were uneven, whereas one-way ANOVA was applied if the variances met the homogeneity criteria. Fisher ‘s least significant difference test was used to compare any two arbitrary groups once statistical significance was established by ANOVA. A threshold of statistical significance was defined as *P* < 0.05.

## Results and discussion

### Selection and validation of differentially expressed genes

**Fig. 2:**
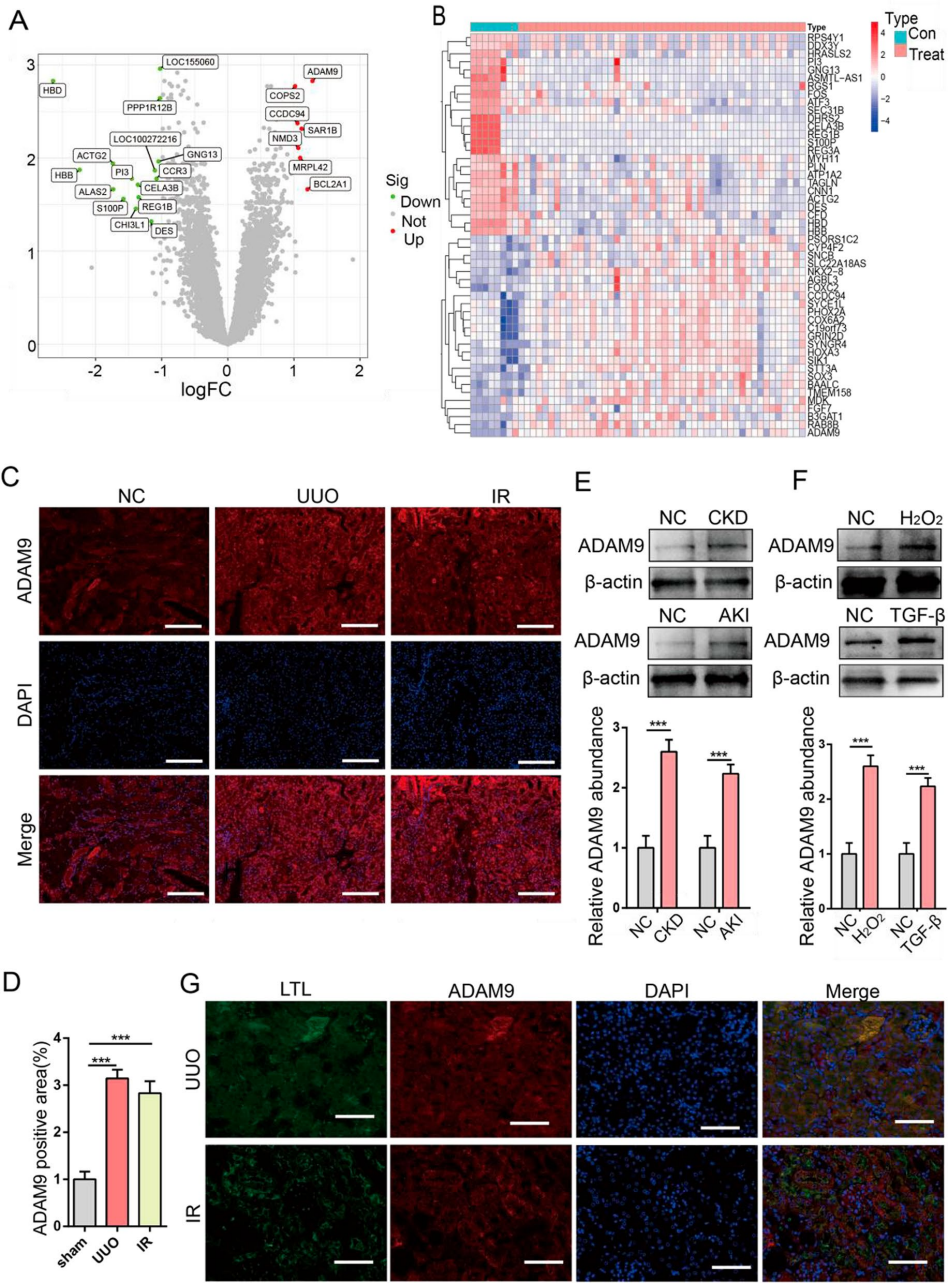
Identification of Differentially Expressed Genes (**A**) Identification of DEGs between the normal group and the chronic kidney disease group using bioinformatics techniques. The results are presented in a volcano plot (*P* < 0.05, |log_2_FC|> 1). (**B**) Heatmap representation of differentially expressed genes (con, healthy controls; treat, CKD patients). (**C** and **D**) Immunofluorescence Detection and quantitative analysis of ADAM9 levels in the kidneys of normal, UUO, and I/R model mice. Magnification: 400 × ; scale bar: 50 μm. (**E**) Western blot detection and quantitative analysis of ADAM9 expression in acute kidney injury (AKI) and chronic kidney disease (CKD). Western blot results and quantitative analysis of ADAM9 levels in renal tubular epithelial cells in H₂O₂- and TGF-β-induced in vitro inflammation and fibrosis models. (**F**) Immunofluorescence co-staining showing the localization of ADAM9 in renal tubular epithelial cells. Magnification: 400 × ; scale bar: 50 μm

Sample information was obtained from the GEO database (GSE66494). The dataset included samples from patients with CKD and healthy controls. We first conducted quality control, alignment, and quantification analyses on the original data to reveal differential gene expression among the different sample groups. We employed rigorous statistical methods to identify genes whose expression differed between the control and experimental groups. A threshold of |log_2_FC|> 1 and a *P* value of < 0.05 were used to determine significance. We further depicted changes in the expression of these genes using a volcano plot and a heatmap for intuitive representations (Figs. 2A and 2B).

To validate the bioinformatics analysis, immunofluorescence assays were performed to detect ADAM9 expression in the kidneys of mice with unilateral ureteral obstruction (UUO) and renal ischemia–reperfusion (I/R). Compared with the NC group, ADAM9 expression was significantly upregulated in the kidneys of UUO and I/R model mice (Figs. 2C and 2D). Western blot analysis was further conducted to examine ADAM9 expression in clinical samples from patients with AKI and CKD. The results were consistent with those from animal models, confirming the overexpression of ADAM9 in clinical specimens (Fig. 2E). For in vitro validation, renal tubular epithelial cells treated with hydrogen peroxide (H₂O₂) were utilized to establish an AKI cell model, whereas cells treated with transforming growth factor-β (TGF-β) were employed to construct a renal fibrosis model. ADAM9 expression in both treated cell models was approximately twice that of normal renal tubular epithelial cells (Fig. 2F).

These findings indicate a positive correlation between ADAM9 expression and both AKI and chronic kidney fibrosis. To determine the cellular localization of ADAM9, immunofluorescence co-staining was performed using antibodies against LTL, a specific marker for renal tubular epithelial cells, and ADAM9. The results demonstrated extensive overlap between the signals of ADAM9 and LTL, confirming ADAM9 expression in renal tubular epithelial cells (Fig. 2G).

### Characterization of M2M, PEI/PLGA, and M2M@NP complexes in vitro

**Fig. 3:**
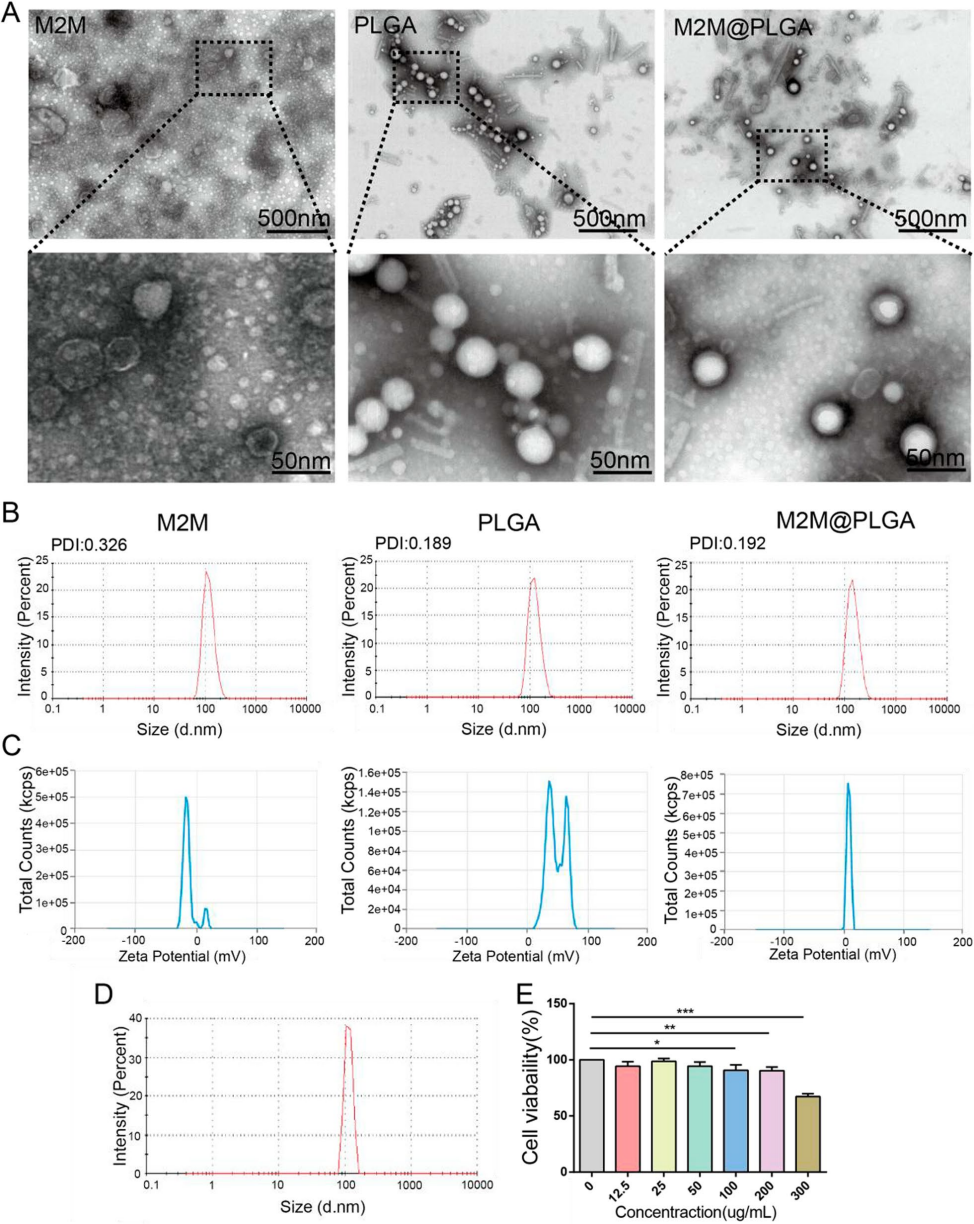
In vitro analysis of M2M, PLGA, and M2M@NP complexes. (**A**) Transmission electron microscopy (TEM) was used to observe M2M, PLGA, and M2M@NP complexes. Scale bars: 50 nm (low magnification) and 500 nm (high magnification). (**B**) Particle size analysis plots of M2M, PLGA, and M2M@NP complexes. (**C**) Zeta potential analysis of M2M, PLGA, and M2M@NP complexes. (**D**) Size and zeta potential analysis of fluorescently labeled PLGA nanoparticles. (**E**) In vitro safety evaluation of nanoparticles. *P < 0.05, ** *P* < 0.01, and ***P < 0.001

The structures of M2M, polyethyleneimine (PEI)-modified PLGA nanoparticles, and the M2M@NP complex were explored by TEM (Fig. 3A). TEM analysis indicated that M2Ms maintained an intact vesicular morphology, whereas the PEI/PLGA nanoparticles exhibited a regular, uniform spherical morphology. By combining them and encapsulating the PEI PLGA nanoparticles with M2M, we developed a new transfection carrier, the M2M@NP complex. PEI/PLGA was wrapped by M2M, forming a spherical vesicle complex with a uniform particle size. The M2M has an average size of 149 nm with a polydispersity index (PDI) of 0.326, the PEI/PLGA nanoparticles exhibit an average size of 156 nm with a PDI of 0.189, and the M2M@NP complex demonstrates an average size of approximately 181.5 nm with a PDI of 0.192 (Fig. 3B). The average zeta potential of M2M was -12.55 mV. PEI-modified PLGA nanoparticles exhibited an average zeta potential of + 48.53 mV, while the M2M@PLGA complex demonstrated an average zeta potential of + 8.84 mV (Fig. 3C). These results indicate that PEI modification confers a positive surface charge to the nanoparticles, facilitating efficient loading of negatively charged M2Ms. Accordingly, the zeta potential increased following PEI modification and subsequently decreased after conjugation with M2Ms. The results showed that the M2M@NP complexes had a more uniform particle size, possibly due to the supporting effect of the PLGA nanoparticles. To evaluate the impact of fluorescent labeling on the physicochemical properties of the nanoparticles, systematic characterization was performed. The labeled nanoparticles maintained an average hydrodynamic diameter of 114 nm, with no significant difference compared to their unlabeled counterparts (Fig. 3D). This confirms that the labeling process effectively preserves the intrinsic characteristics of the nanoparticles. Furthermore, the concentration-dependent cytotoxicity of PLGA nanoparticles toward renal tubular epithelial cells was evaluated. The cells were treated with a concentration gradient of nanoparticles (0–300 μg/mL) for 24 h, and cell viability was quantitatively assessed using the CCK-8 assay. Notably, PLGA nanoparticle concentrations up to 50 μg/mL demonstrated no significant adverse effects on cell viability (Fig. 3E).

**Fig. 4:**
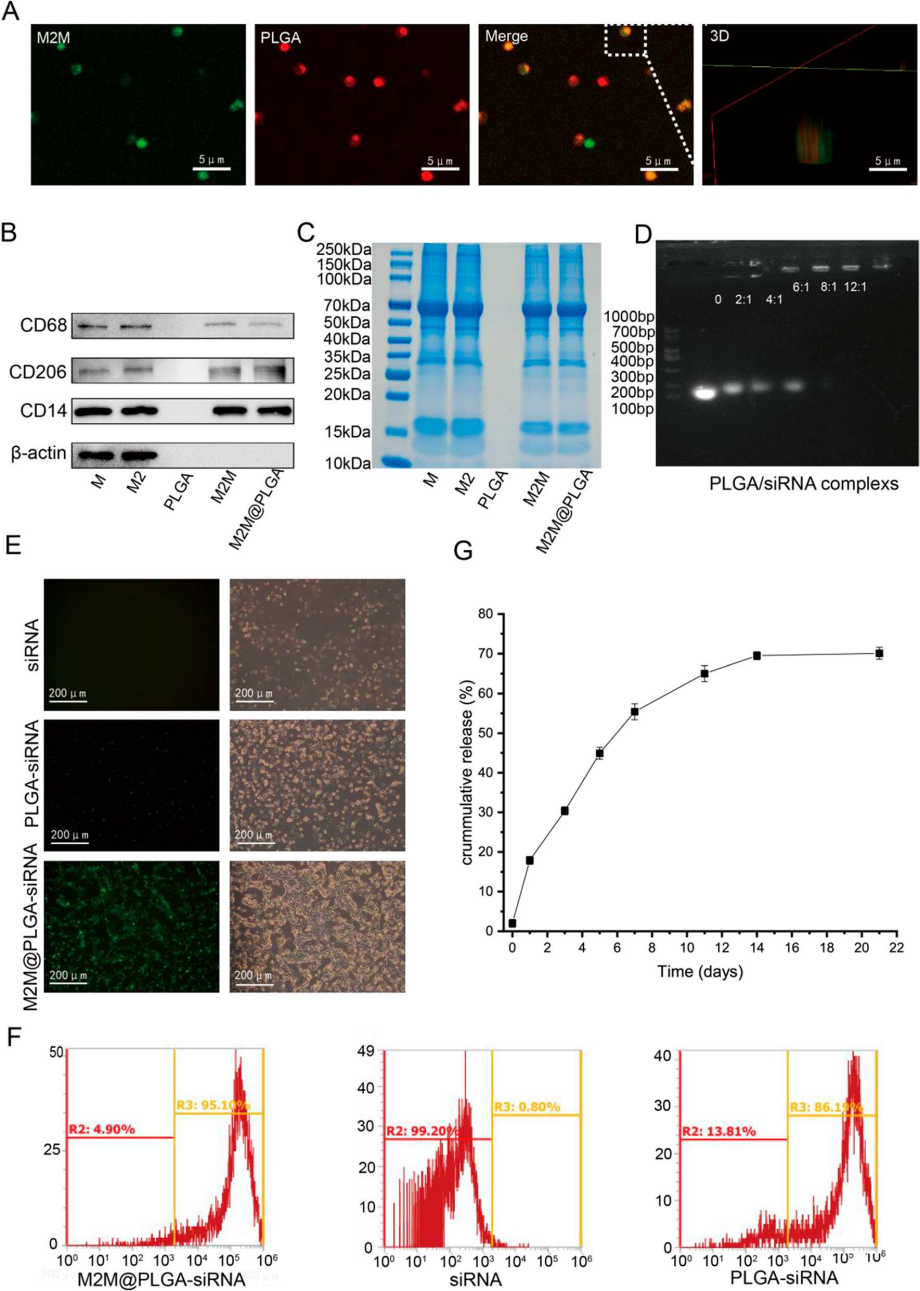
(**A**) Fluorescence images of PLGA and M2M and a merged image of M2M@NPs from three-dimensional analysis. (**B**) Western blot analysis of key proteins expressed by M2Ms. (**C**) Coomassie brilliant blue staining of M2M, PLGA, and M2M@NPs. (**D**) Agarose gel electrophoresis of complexes with varying N/M ratios (N: PEI-PLGA nanoparticles; M: siRNAs). (**E**) Transfection images showing the uptake of siRNA into renal tubular epithelial cells. (**F**) Transfection efficiency analysis. (**G**) In vitro cumulative release profile of siRNA from siRNA NP complexes in PBS (pH 7.2)

Red rhodamine B dye was used to label PEI/PLGA, whereas M2M was labeled with FITC to observe the formation of the M2M@NP complex. The fluorescence localization results clearly demonstrated the positions of M2M, PEI/PLGA, and the M2M@PLGA complex. Further three-dimensional imaging revealed that the red fluorescence and green fluorescence in a single M2M@NP overlapped well (Fig. 4A). We extracted M0 macrophages, M2 macrophages, M2Ms, PEI/PLGA, and the M2M@NP complex to verify whether M2M@NPs retained the essential proteins in M2Ms. We detected three characteristic membrane markers (CD68, CD206, and CD14) and an intracellular marker (GD) on the M2 macrophage membrane using Western blotting (Fig. 4B). Western blot analysis revealed that M2M and M2M@PLGA retained marker proteins on the M2 macrophage membrane but did not retain the nuclear proteins of M2 macrophages. Coomassie brilliant blue staining revealed a protein band at approximately 70 kDa. The protein band in the M2 group was more intense than that in the M2M group, whereas the protein bands in the M2M and M2M@PLGA groups were of similar intensity (Fig. 4C). These results indicate that the biological function of the macrophage membrane was maintained in the M2M@NP complex. PEI/PLGA (56 µg) and 56 µg of siRNA were mixed and incubated for 25 min. The mixture was then added to a six-well plate containing renal tubular epithelial cells, with each well receiving 16 µg of the complex. Subsequently, gel shift assays confirmed that the nanoparticles fully encapsulated the siRNA at an N/P ratio of 6:1 (the molar ratio of PEI amine to DNA phosphate) (Fig. 4D). Renal tubular epithelial cells were transfected with fluorescein-labeled siRNA, PEI/PLGA-siRNA complexes, and M2M@PLGA-siRNA complexes. Transfection efficiency was evaluated 6 h post-transfection by assessing green fluorescence intensity and performing flow cytometric analysis. Cells transfected with naked siRNA exhibited almost no detectable green fluorescence, with a transfection efficiency of approximately 0.8%. In contrast, PEI/PLGA-siRNA transfection produced moderately increased fluorescence intensity, achieving a transfection efficiency of 86.19%. Notably, transfection with M2M@PLGA-siRNA resulted in significantly enhanced green fluorescence signals, with a transfection efficiency of 95.10%, indicating that the M2M@PLGA delivery system efficiently delivers siRNA into renal tubular epithelial cells (Figs. 4E and 4F). In addition, a sustained release profile of siRNA from the PMVs@siRNA NP complexes was observed, with approximately 80% of the loaded siRNA released over a 14-day period (Fig. 4G).

Collectively, these results demonstrate that M2M@PLGA complexes represent an effective transfection vector for both in vivo and ex vivo applications, attributable to their uniform particle size, high transfection efficiency, retention of key macrophage membrane proteins, and favorable safety profile with non-toxicity.

### Transcriptome sequencing

**Fig. 5:**
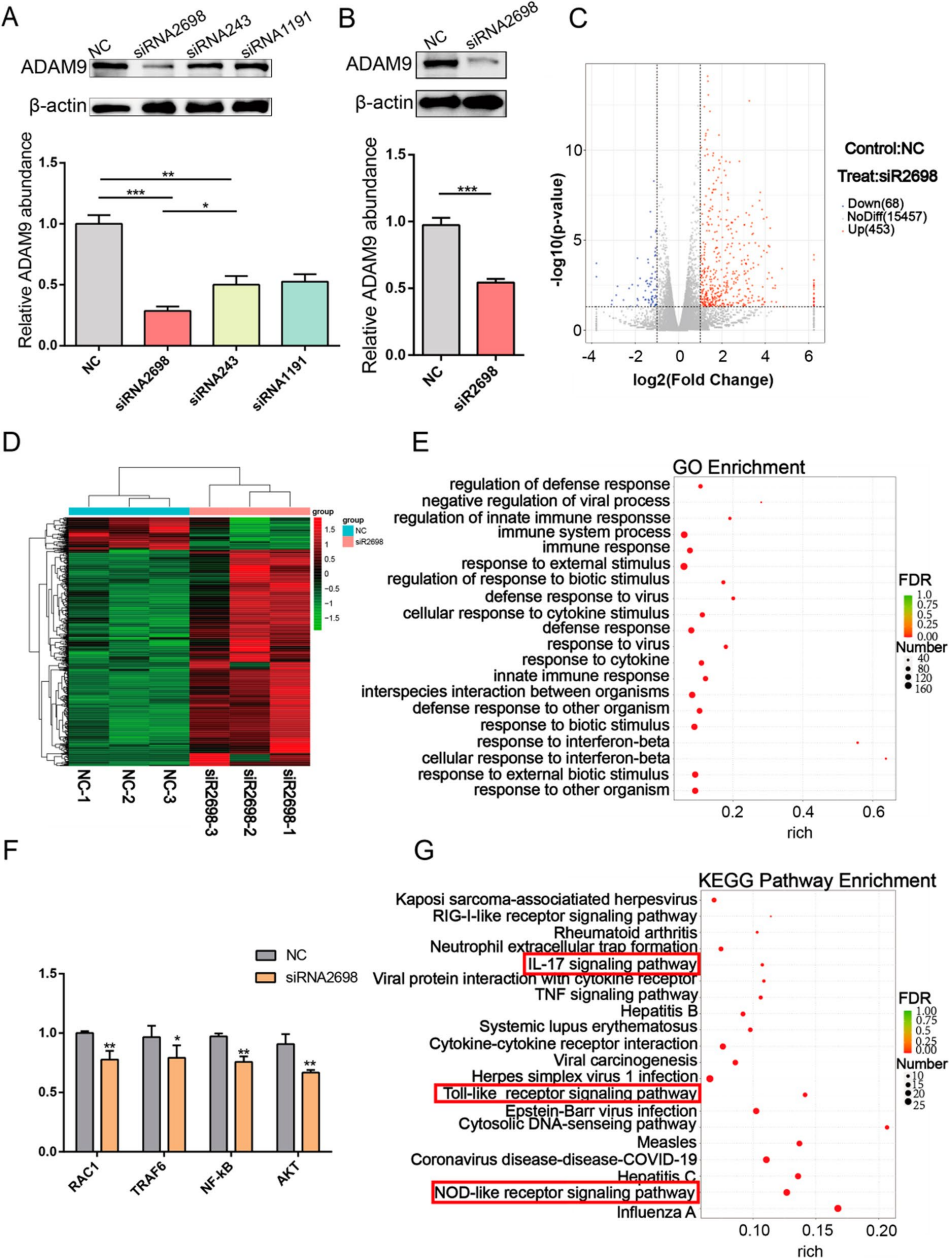
Transcriptome Sequencing. (**A** and **B**) Renal tubular epithelial cells were transfected with four siRNAs, followed by Western blot analysis to determine and validate ADAM9 expression. (**C**) Volcano plot illustrating DEGs between the NC group and the siRNA2698 group. *P* < 0.05, |log_2_FC|> 1. (**D**) Heatmap illustrating DEGs between the NC group and the siRNA2698 group. (**E**) GO pathway enrichment analysis of the transcriptome sequencing data. (**F**) Quantitative analysis of inflammatory gene expression levels in the normal and ADAM9-knockdown groups. (**G**) KEGG pathway enrichment analysis of the transcriptome sequencing data

Proteins were extracted, and Western blotting analysis revealed that M2M@NP-2698 siRNA successfully reduced ADAM9 expression (Figs. 5A and 5B). These findings validate the potential of this siRNA construct for targeting ADAM9 for therapeutic purposes. We performed differential gene expression analysis on transcriptomic data from the normal group and the ADAM9-knockdown group. A volcano plot was used to visualize the relationship between the log_2_FC values of all DEGs and their statistical significance, as well as the expression changes of all genes between the control and experimental groups (Fig. 5C). We conducted expression profile clustering analysis and created a heatmap to visualize the overall expression pattern differences among samples under different conditions (Fig. 5D). The heatmap clearly showed distinct separation between the normal and knockdown groups in terms of gene expression levels, indicating that knockdown had a significant impact on the global transcriptional landscape. The biological processes, molecular functions, and cellular components significantly affected by the knockdown of a specific gene, as well as the major signaling pathways involved, were revealed through GO and KEGG pathway functional enrichment analysis (Figs. 5E and 5G). We found that these DEGs were primarily associated with pathways related to inflammation, the TLR signaling pathway, the IL-17 signaling pathway, and the NOD-like receptor signaling pathway. Among these genes, several DEGs closely related to inflammatory responses, including TRAF6, RAC1, NF-κB, and AKT, were selected for further investigation (Fig. 5F). The TLR pathway typically activates the NF-κB signaling cascade, leading to the robust release of pro-inflammatory cytokines such as TNF-α and IL-1β. In the rosacea inflammation model, ADAM9 expression was significantly upregulated in LL37-induced HaCaT cells, which further promoted the increased expression of proteins in the ADAM9/TLR2/NF-κB P65 pathway, thereby exacerbating the inflammatory response [36]. In contrast, ADAM9 silencing markedly inhibited this ADAM9-mediated TLR2/NF-κB inflammatory signaling pathway, effectively blocking the excessive inflammatory cascade triggered by the TLR pathway. Moreover, ADAM9 silencing enhanced the phagocytic capacity of macrophages for apoptotic neutrophils. Impaired clearance of apoptotic cells can activate the NOD-like receptor pathway, suggesting that ADAM9 silencing may indirectly modulate inflammation through improved apoptotic cell clearance [37, 38]. Additionally, ADAM9 regulates Th17 cell function via metabolic pathways. In Adam9⁻/⁻ mouse models, silencing ADAM9 inhibited GLUT1 expression and glycolysis in Th17 cells, resulting in impaired cell differentiation, reduced infiltration of CD4⁺ T cells in the kidney, and attenuated IL-17-related inflammatory signaling. These effects ultimately alleviate inflammatory damage in the kidney and other organs [39].

### The mechanism of M2M@NP-ADAM9 siRNA in alleviating renal inflammation

**Fig. 6:**
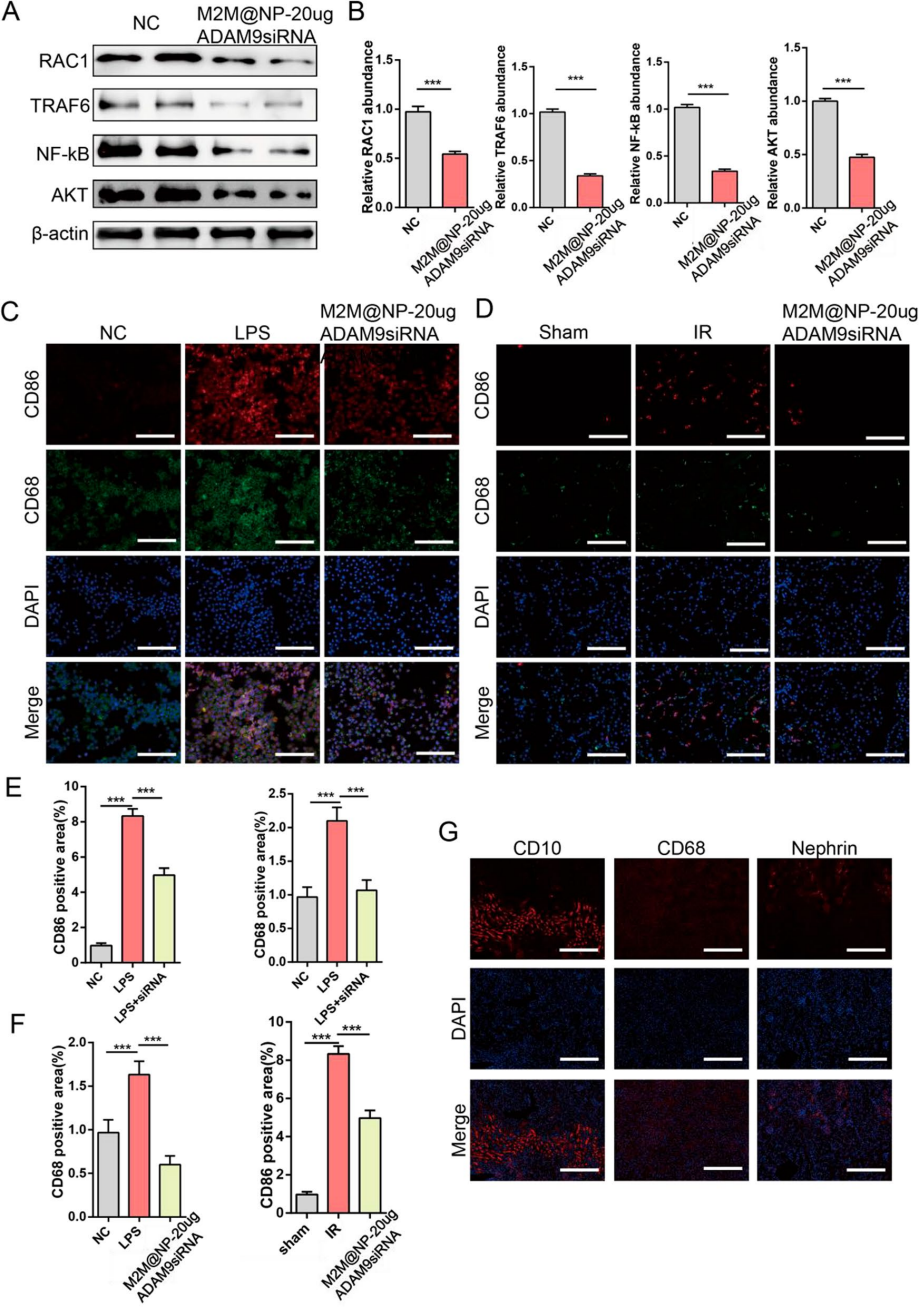
(**A** and **B**) Western blotting results of inflammation-related genes at the protein level and quantitative analysis. (**C**) Immunofluorescence staining of CD86 and CD68 in the kidney tissue of I/R mice. Magnification: 400 × ; scale bar: 50 μm. (**D**) Immunofluorescence staining for CD86 and CD68 expression in macrophages. ADAM9 siRNA alleviated LPS-induced M1 polarization in macrophages. RAW264.7 cells were exposed to LPS and ADAM9 siRNA. After 24 h of treatment, the cells were subjected to immunofluorescence analysis. Magnification: 400 × ; scale bar: 50 μm. (**E** and **F**) The relative immunofluorescence intensities of CD86 and CD68. (**G**) Immunofluorescence images demonstrating the specific targeting of M2M@PLGA to multiple cell types within the renal inflammatory microenvironment. Magnification: 400 × ; scale bar: 50 μm

The protein expression levels of these genes were re-evaluated to further validate the reliability of the transcriptome sequencing results. The Western blot results confirmed that the gene expression levels aligned with the transcriptome sequencing results, confirming the effect of ADAM9A knockdown on the expression of inflammation-related genes (Figs. 6A and 6B). We treated RAW264.7 cells with lipopolysaccharide (LPS) to establish an inflammatory cell model and observe the regulatory effect of ADAM9 on macrophage polarization in vivo. The characteristic macrophage phenotypes indicated that the percentage of CD86 + CD68 + macrophages significantly increased in the LPS-treated group compared with the control group, suggesting an increase in macrophage numbers and polarization toward the M1 phenotype. However, increases in the percentage of CD86 + CD68 + macrophages were mitigated in LPS-treated macrophages transfected with ADAM9 (Figs. 6C and 6E). Changes in the M1 phenotype were confirmed by immunofluorescence staining of tissue sections from I/R mice (Figs. 6D and 6F). The immunofluorescence results suggested that the quantity of CD86 + CD68 + macrophages was higher in I/R mice than in control mice. After treatment with ADAM9 siRNA, the increase in CD86 + CD68 + macrophages in mice was significantly reduced. These results demonstrate that inflammation can lead to the accumulation of macrophages and M1 polarization, whereas ADAM9 siRNA alleviates M1 polarization, thereby reducing inflammation. To identify the cellular targets of M2M@PLGA, a murine model of bilateral renal I/R injury was established. Fluorescently labeled M2M@PLGA was administered via tail vein injection, and kidney tissues were collected for multi-marker immunofluorescence analysis. Significant co-localization was observed between M2M@PLGA and CD10 (proximal tubular cells), CD68 (macrophages), and Nephrin (podocytes), demonstrating that M2M@PLGA can simultaneously target multiple cell types within the inflammatory microenvironment.

### In Vivo targeting of injured In Vivo areas

**Fig. 7:**
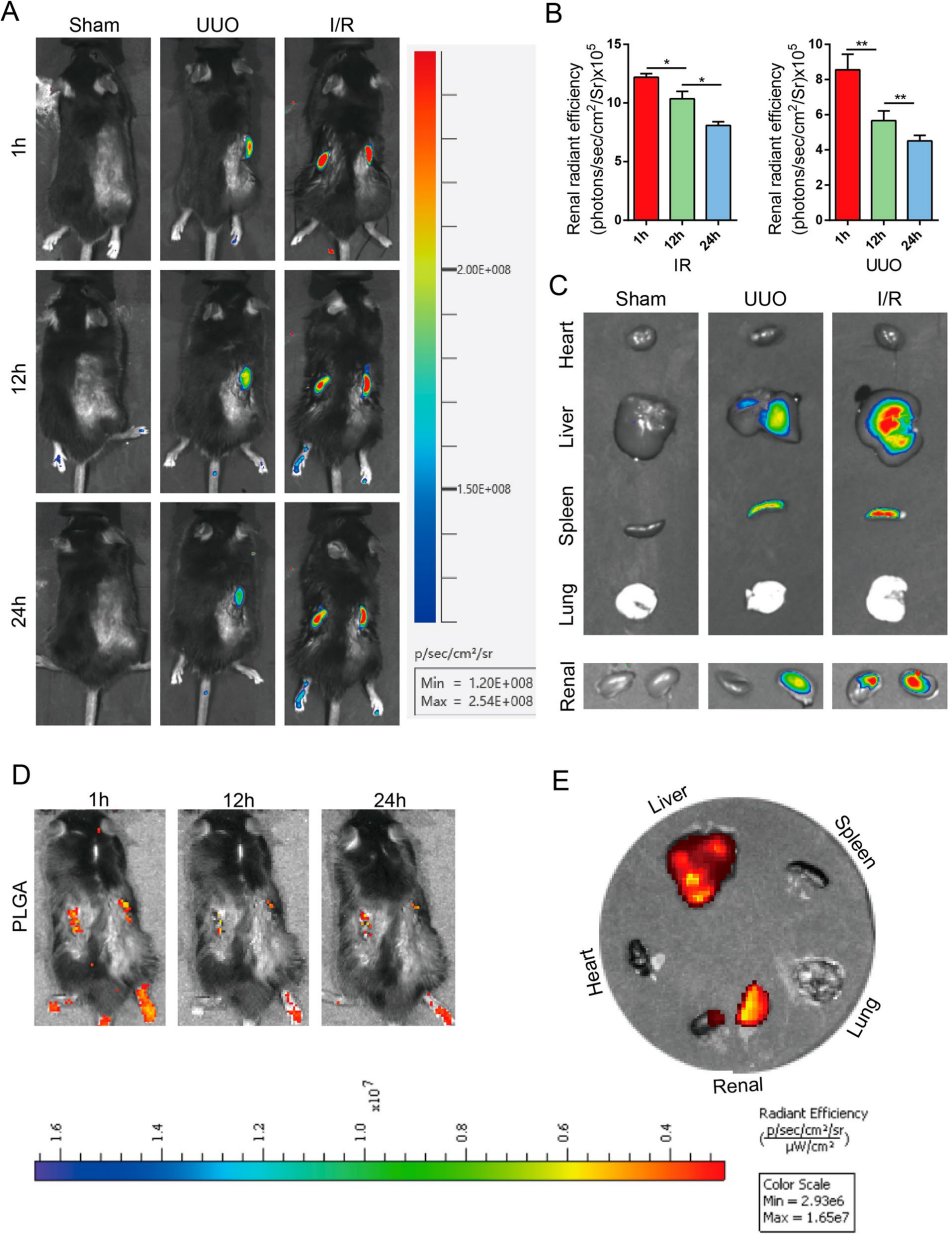
Fluorescence imaging results of M2M@NPs in the UUO and I/R groups. (**A** and **B**) Optical fluorescence imaging results of DiR-labeled M2M@NPs in the sham, UUO, and I/R groups at various time intervals following injection. (**C**) Ex vivo fluorescence images of five major tissues in the sham, UUO, and I/R groups at multiple time points after intravenous administration. (**D**) Fluorescence imaging of the I/R group at multiple time points following injection of DiR-labeled PLGA nanospheres. (**E**) Ex vivo fluorescence imaging of five major tissues in the I/R group at multiple time points following intravenous administration of PLGA nanoparticles

PEI/PLGA nanoparticles were labeled with DiR and then mixed with M2M to form M2M@NP^Dir^ complexes. Experiments were conducted in IR and UUO mouse models to investigate the effects of these complexes, with normal mice serving as controls. The mice received intravenous tail injections of the labeled M2M@NP complexes, and fluorescence imaging changes at different time points were observed (Fig. 7A). In vivo imaging analysis revealed that the M2M@NP complexes had significant targeting specificity toward injured kidneys (Fig. 7B). Ex vivo imaging of injured renal tissue revealed similar results, suggesting that a simple tail vein injection of the M2M@NP delivery system effectively directed siRNA to injured kidneys, providing a novel therapeutic concept for AKI and chronic fibrosis. Furthermore, ex vivo imaging revealed that the M2M@NP complexes were primarily metabolized by the liver and kidneys, while the heart, spleen, and lungs were all imaged (Fig. 7C). To exclude nonspecific renal accumulation of PLGA nanoparticles caused by potential M2M leakage, fluorescently labeled PLGA nanoparticles were administered via tail vein injection for comparative imaging analysis. Both in vivo real-time imaging and ex vivo organ-specific evaluation demonstrated substantially diminished fluorescence signals in the kidneys. (Figs. 7D and 7E).

### Biosafety assessment

**Fig. 8:**
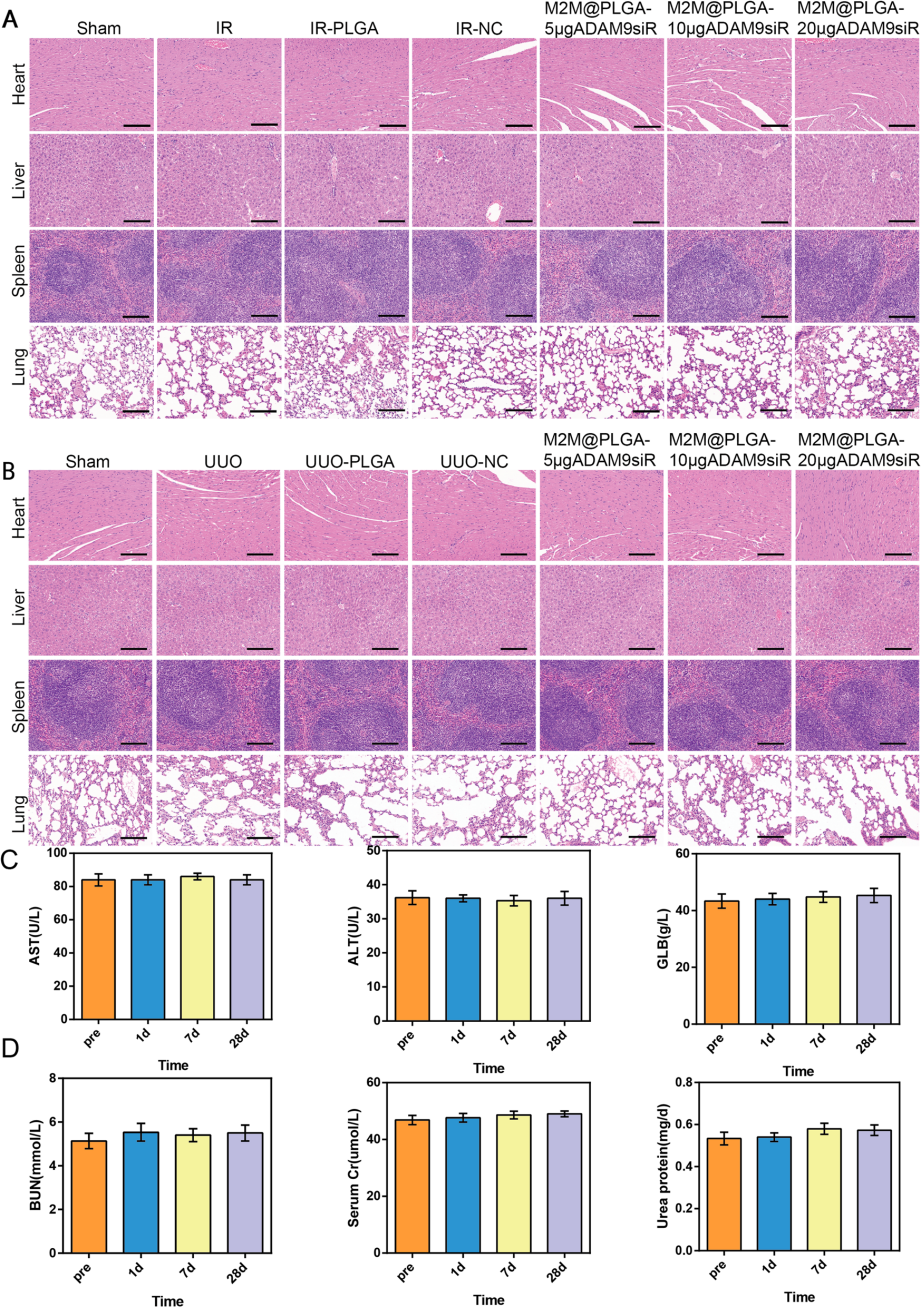
Biosafety of M2M@NPs. (**A** and **B**) H&E-stained images of major tissues except for the kidneys in I/R and UUO mice. Magnification: 400 × , scale bar: 100 μm. (**C** and **D**) Bar charts illustrating hepatic and renal function parameters in mice at four time points

The UUO model mice in the IR groups were euthanized at weeks 1 and 3. Sections of multiple organs except for the kidneys were stained with H&E. The results revealed no significant differences among the M2M@NP, M2M@NP-NC siRNA, various concentrations of M2M@NP-ADAM9 siRNA, control, and untreated model groups (Figs. 8A and 8B). These findings indicate the significant biosafety of the M2M@NP delivery system. Then, we confirmed the aforementioned perspective by analyzing biochemical blood indicators. We collected blood samples for analysis before injecting M2M@NP-ADAM9 siRNA, as well as on days 1, 7, and 28 post-injection. The experimental findings indicated that blood biochemical parameters, such as liver function markers (aspartate aminotransferase, alanine aminotransferase, and globulin) and kidney function markers (blood urea nitrogen (BUN), creatinine (Cr), and urea), did not significantly differ across the four groups (Figs. 8C and 8D).

### Effects of M2M@NP-ADAM9 siRNA on renal I/R injury In Vivo

**Fig. 9:**
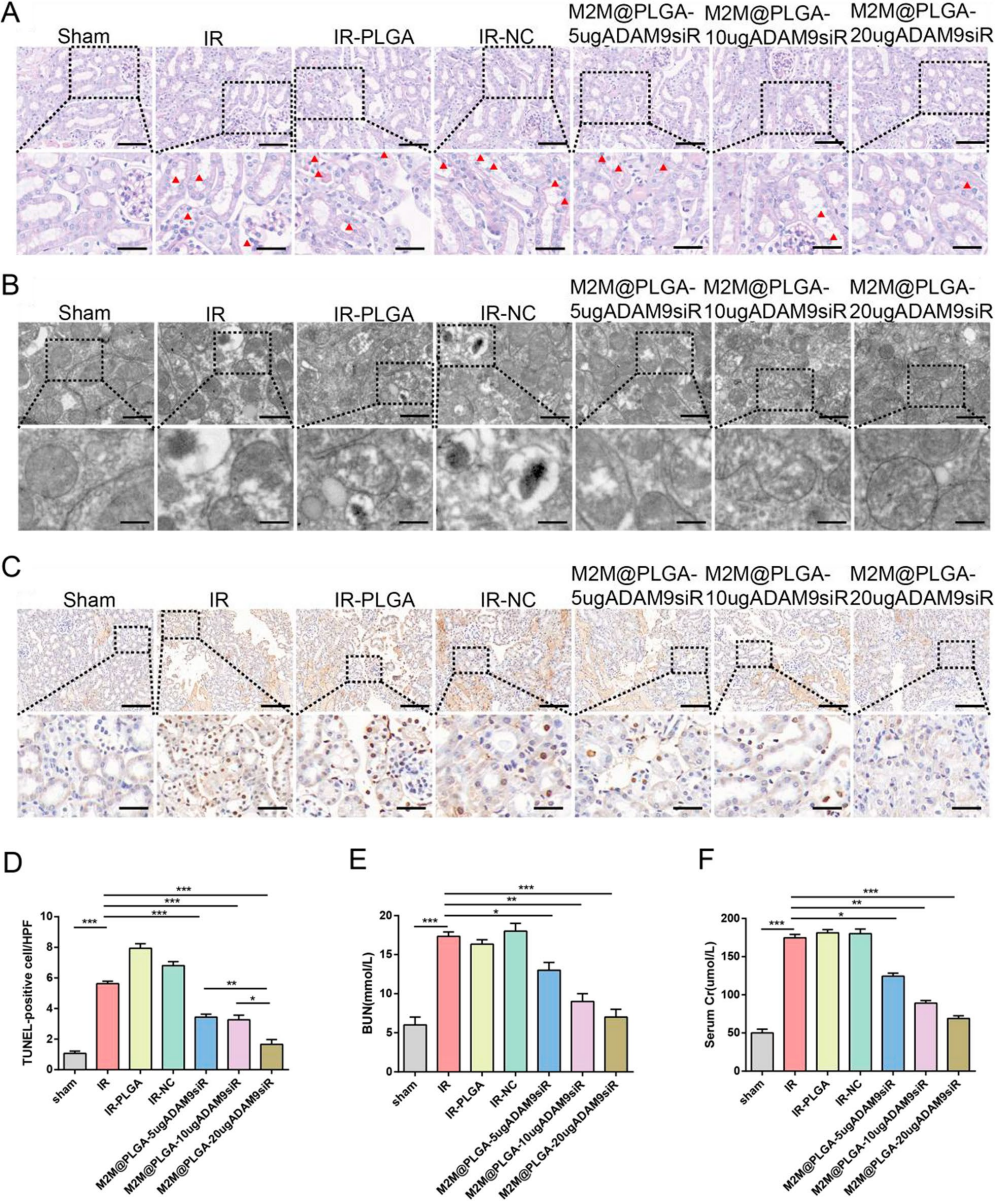
(**A**) PAS-stained sections of kidney tissue. The red arrowheads denote the swelling of renal tubular epithelial cells and nuclear detachment. Scale bars: 50 μm (× 200) and 200 μm (× 400). (**B**) Electron micrograph of mitochondria observed in kidney tissue using transmission electron microscopy (TEM). Scale bars: 0.2 μm (× 400) and 1 μm (× 2000). (**C**) Images of renal tubules observed by TUNEL staining. Scale bars: 50 μm (× 200) and 200 μm (× 400). (**D**) Quantification of TUNEL staining results. (**E** and **F**) BUN and SCr levels were measured 72 h after M2M@NP treatment

As discussed, M2M@NP-ADAM9 siRNA enhanced the delivery of ADAM9 siRNA to damaged kidney tissue, which is a prerequisite for treating renal injury. The main pathological alterations observed in I/R, such as the enlargement of renal tubular epithelial cells, cellular degeneration, cell death, detachment, infiltration of inflammatory cells, and even sloughed-off nuclei and tubular casts visible within the renal tubular lumina, were particularly evident in the I/R, I/R-PLGA, and I/R-NC groups of mice in the bilateral I/R model, as assessed by periodic acid–Schiff staining. These pathological alterations (indicated by red arrows) were alleviated to varying degrees in the groups treated with different drug concentrations (Fig. 9A). Our previous research revealed that mitochondrial damage is an essential factor in the development of AKI. Using TEM, we observed mitochondria in the kidney cortex of mice at 24 h post-treatment (Fig. 9B). More autophagosomes and autolysosomes were observed in the I/R, I/R-PLGA, and I/R-NC groups than in the sham group. Decreases in mitochondrial vacuolization and fewer cristae fractures were observed following treatment with various concentrations of M2M@NP-ADAM9 siRNA. We also performed TUNEL assays, which revealed a significantly lower ratio of TUNEL-positive cells in kidneys treated with different concentrations of M2M@NP-ADAM9 siRNA than in the untreated I/R group (*P* < 0.001) (Figs. 9C and 9D). We measured BUN and sCr levels after administering M2M@NP-ADAM9 siRNA via intravenous injection to the I/R model mice to demonstrate the efficacy of M2M@NP-ADAM9 siRNA in treating ischemic AKI (Figs. 9E and 9F). Compared with the sham group, the I/R, I/R-PLGA, and I/R-NC groups showed significantly elevated BUN and sCr levels, whereas the M2M@NP-ADAM9 siRNA group substantially reduced BUN and sCr levels in I/R mice (*P* < 0.001). The data clearly show that M2M@NP-ADAM9 siRNA mitigated renal damage induced by I/R.

### M2M@NP-ADAM9 siRNA treatment decreases renal inflammation

**Fig. 10:**
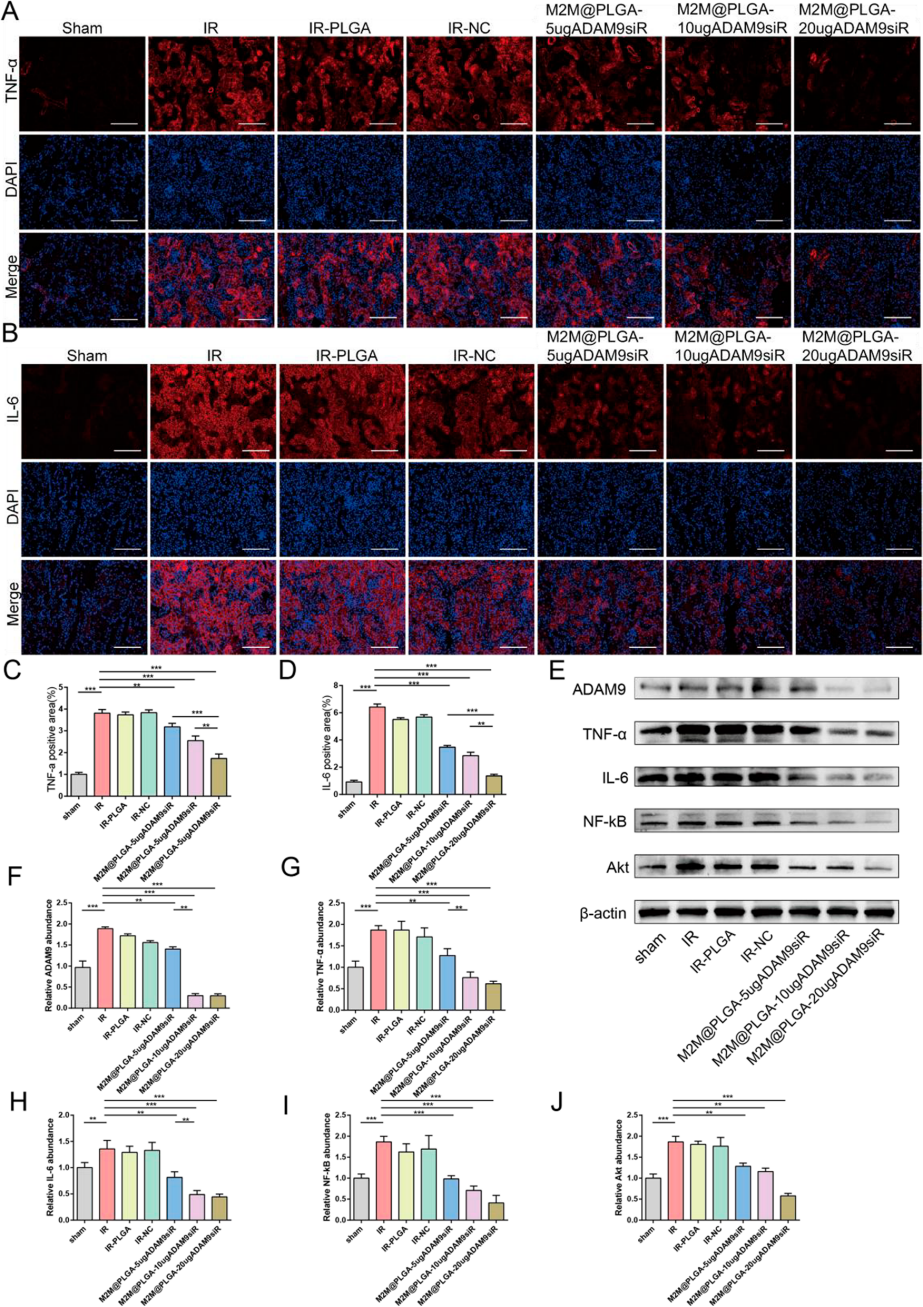
(**A** and **B**) Immunofluorescence staining of TNF-α and IL-6 in the kidney tissue of I/R mice. Magnification: 400 × ; scale bar: 50 μm. (**C**-**D**) Quantification of TNF-ɑ and IL-6 levels. (**E**) Western blot results for marker proteins, including ADAM9, TNF-ɑ, IL-6, NF-kB, and AKT. (**F**-**J**) Quantification of ADAM9, TNF-ɑ, IL-6, NF-κB, and AKT expression. *P < 0.05, ** *P* < 0.01, and ***P < 0.001

We assessed the inflammatory response in injured kidneys by measuring the expression levels of inflammatory molecules, including TNF-a and IL-6. Immunofluorescence analysis and Western blotting confirmed that TNF-a and IL-6 expression were increased in the I/R, I/R-PLGA, and I/R-NC groups but were reduced in the 5 μg ADAM9 siRNA, 10 μg ADAM9 siRNA, and 20 μg ADAM9 siRNA groups (Fig. 10A-H). These results indicate that the M2M@NP-ADAM9 siRNA delivery system effectively decreased the inflammatory response during renal repair. We monitored changes in the phosphorylation levels of NF-κB and Akt, which are primary markers of pathway activity, to explore the impact of ADAM9 gene knockdown on downstream inflammatory signaling pathways. The Western blot results demonstrated that the protein expression levels of NF-κB and Akt decreased with increasing treatment concentrations (Fig. 10E). The decreases were statistically significant (*P* < 0.05) (Figs. 10I and J). These results show that ADAM9 might regulate TNF-α and IL-6 levels through these signaling pathways, thereby alleviating the inflammatory response.

### Attenuation of renal fibrosis in UUO mice

**Fig. 11:**
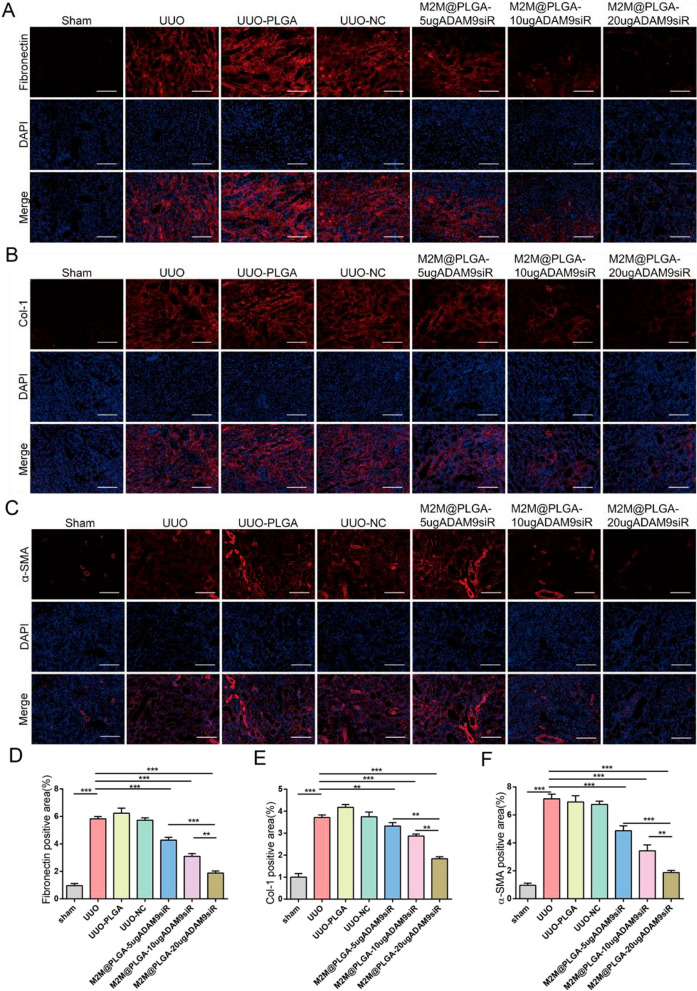
(**A**-**C**) Immunofluorescence images of Fn, Col-1, and α-SMA in the kidney tissues of mice from each UUO model group. Magnification: 400 × ; scale bar: 50 μm. (**D**-**F**) Quantification of Fn, Col-1, and α-SMA levels

As renal fibrosis progresses, kidney function gradually declines, potentially leading to renal failure. Therefore, slowing or halting the development of kidney fibrosis is crucial for delaying the progression of CKD and improving patient outcomes [66]. We explored the effects of M2M@NP-ADAM9 siRNA on renal fibrosis in mice. Fibronectin and type I collagen collectively maintain the stability and functionality of the extracellular matrix through their unique biochemical and physical properties. Under pathological conditions, the abnormal expression and deposition of these two proteins can lead to changes in tissue structure and functional decline, thereby affecting the health and function of organs [68, 69]. Thus, immunofluorescence staining for fibronectin and type I collagen was performed on tissue sections (Figs. 11A and B), and the results were quantitatively analyzed (Figs. 11D and E). The levels of fibronectin and type I collagen were significantly increased in the UUO, UUO-PLGA, and UUO-NC groups but notably decreased in the M2M@NP-ADAM9 siRNA group. Myofibroblasts are closely associated with the formation of the extracellular matrix and lead to renal interstitial fibrosis. We also conducted immunofluorescence staining for α-SMA, a marker protein for myofibroblasts (Fig. 11C). Compared with the sham group, the positively stained interstitial areas were significantly larger in the UUO, UUO-PLGA, and UUO-NC groups. However, the positively stained areas gradually decreased with increasing M2M@NP-ADAM9 siRNA concentrations (Fig. 11F). These findings indicate that M2M@NP-ADAM9 siRNA can alleviate the excessive deposition of the extracellular matrix caused by the overactivity of α-SMA, thereby slowing the development of kidney fibrosis.

**Fig. 12:**
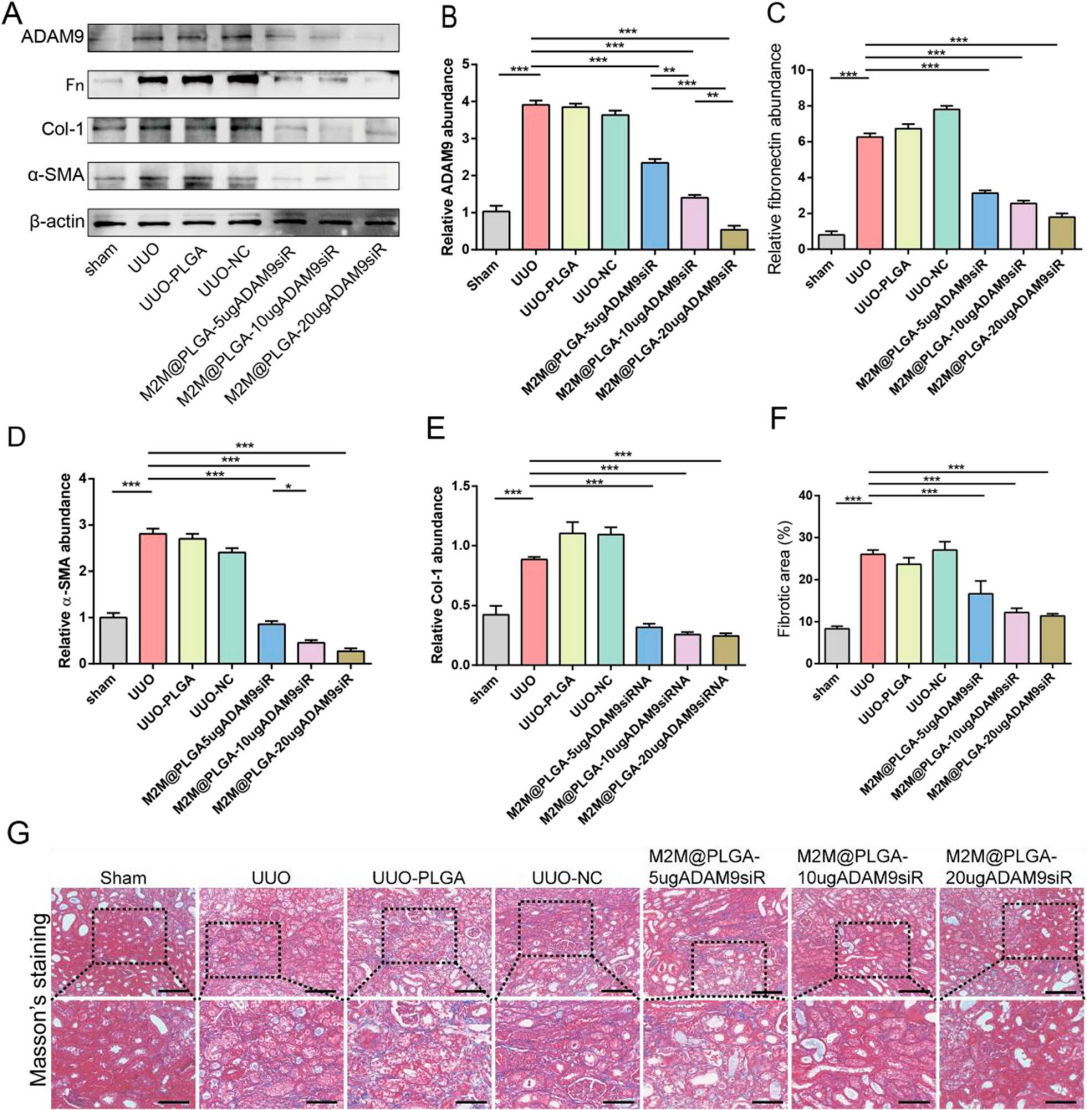
(**A**) Western blot results demonstrating the expression of marker proteins, including ADAM9, Fn, Col-1, and α-SMA. (**B**-**E**) Quantification of ADAM9, Fn, Col-1, and ɑ-SMA expression. *P < 0.05, ** *P* < 0.01, and ***P < 0.001. (**F**) Quantitative analysis of Masson staining. (**G**) Masson-stained images of kidney tissue samples from UUO mice. Scale bars: 50 μm (× 200) and 200 μm (× 400)7

We also corroborated these findings by evaluating the expression of fibronectin, type I collagen, and α-SMA in renal tissues via Western blotting (Fig. 12A) and quantitative analysis (Fig. 12B-E). Kidney tissue sections from each UUO model mouse on day 14 were subsequently stained with Masson ‘s trichrome (Fig. 12G). Larger areas of blue-stained renal interstitium were observed in the untreated UUO group than in the sham group, and the different treatment groups showed a gradual reduction in the area of blue staining with increasing drug concentration. We also quantitatively assessed the percentage of blue-stained areas in the total field of view (Fig. 12F). The area of blue staining in the renal interstitium was lower in the M2M@NP-ADAM9 siRNA group than in the other groups (*P* < 0.01), suggesting that M2M@NP-ADAM9 siRNA could reduce damage to the tubulointerstitium since it significantly decreased renal interstitial fibrosis.

## Discussion

The burden of CKD imposes a substantial impact on disability, shortens life expectancy, and causes many deaths each year [40]. Thus, finding innovative approaches to treat CKD is particularly important. Bioinformatics analysis identified the significantly different genes between CKD patients and healthy individuals. Among these, ADAM proteins perform multiple biological functions, such as the development of inflammation, tissue repair, and cell‒cell interactions [41–44]. ADAM9 (also known as metalloproteinase/disintegrin/cysteine-rich protein 9 (MDC9) or meltrin-γ) is a member of the ADAM family. It was first discovered in breast cancer in 1996 [45] and contributes to a range of pathological states, including inflammation and the development of cancer [46, 47]. The ADAM9 protein is primarily found in tertiary gelatinous granules and secretory vesicles. Pro-inflammatory factors can induce polymorphonuclear leukocyte (PMN) degranulation, thereby increasing the level of ADAM9 on the PMN surface [18]. ADAM9 is involved in cell‒cell and cell‒matrix interactions and may promote tissue repair following injury, as well as the accumulation of the extracellular matrix [21]. Studies have shown that reducing the expression of ADAM9 can alleviate the upregulation of TNF-α, IL-6R, VE-cadherin, and CX3CL1 in human aortic smooth muscle cells (HASMCs) and human aortic endothelial cells (hAoECs), thereby alleviating inflammation. It can also reverse the infiltration of macrophages, thus slowing the progression of abdominal aortic aneurysm (AAA) [48]. Based on these findings, we speculate that ADAM9 may play a role in the development of AKI and CKD. Consistently, experimental results demonstrated that ADAM9 was significantly upregulated in both I/R and UUO mouse models. ADAM9 deficiency attenuated TRAF6 ubiquitination and reduced Rac1-GTP loading, thereby suppressing the TRAF6-Rac1 signaling cascade. This inhibition led to diminished macrophage infiltration, reduced M1 macrophage polarization, and alleviated inflammatory responses. The decreased TRAF6 activity further impaired nuclear translocation of the NF-κB p65 subunit and downregulated NF-κB-dependent AKT phosphorylation. Collectively, these molecular events decreased transcription of pro-inflammatory cytokines (e.g., TNF-α and IL-6), suppressed the fibroblast-to-myofibroblast transition (as indicated by reduced α-SMA expression), and inhibited collagen I synthesis. Ultimately, these coordinated mechanisms effectively ameliorated AKI and chronic tubulointerstitial fibrosis. This finding allows us to further explore how reducing ADAM9 expression can block the expression of multiple inflammation-related pathways, reduce macrophage infiltration, and decrease macrophage M1 polarization. However, long-term silencing of ADAM9 leads to excessive suppression of inflammatory responses, resulting in imbalanced macrophage polarization at the injury site. Although ADAM9 promotes the release of pro-inflammatory cytokines (e.g., TNF-α and IL-6), it also mediates signaling pathways involved in inflammation resolution and tissue repair. Consequently, while the pro-inflammatory activity of M1 macrophages is suppressed, the tissue repair functions of M2 macrophages are impaired under ADAM9 deficiency. Macrophages regulate renal tissue regeneration through the secretion of factors such as transforming growth factor-β (TGF-β) and hepatocyte growth factor (HGF); however, long-term silencing of ADAM9 disrupts this regulatory network, causing asynchrony between inflammation clearance and the initiation of repair processes, ultimately delaying renal tissue regeneration [37, 49]. By expressing TLRs, macrophages play a central role in innate immunity and serve as the primary defense against infections. Macrophages interact with various inflammatory factors, making them key cells in chronic inflammation and related pathologic processes [50]. Our combined experimental findings suggest that ADAM9 may directly alleviate chronic renal tubulointerstitial fibrosis by reducing macrophage infiltration. M1 macrophages have pro-inflammatory effects [51, 52]. Kidneys that recover from AKI may exhibit chronic dysfunction, tubular atrophy, and interstitial fibrosis, with some cases progressing to CKD. Reducing the expression of ADAM9 can alleviate inflammation and injury caused by AKI, promote repair, and slow the onset of CKD [53–55].

However, effective intervention with ADAM9 in vivo* is challenging*. Our cellular experiments confirmed that ADAM9 siRNA could effectively interfere with ADAM9. However, direct tail vein injections of siRNA into mice do not precisely target the damaged area and have a short half-life. Therefore, we considered encapsulating the siRNA in nanoparticles covered with a macrophage membrane, utilizing the ability of the macrophage membrane to target the damaged kidney. The M2 macrophage membrane may target inflamed areas [56]. This system transfers bioactive cell membranes onto the exterior of the nanoparticles while maintaining excellent biocompatibility and preserving the functionality of the macrophage membrane along with the superior physical properties of conventional nanoparticles [27, 57]. In this study, PEI-functionalized PLGA was selected to achieve electrostatic adsorption of siRNA, with PEI/PLGA serving as the structural core. High-molecular-weight PEI (e.g., 25 kDa) has a strong cationic density that can disrupt cell membrane integrity, induce apoptosis, and, due to its non-degradable backbone, accumulate in vivo, leading to long-term toxicity. Studies have shown that unmodified PEI at concentrations ≥ 2.5 μM reduces the survival of cells such as HCT116 and HepG2 to below 50%, whereas PEI modified with PLGA reduces cytotoxicity by more than 80% [58]. PEI-siRNA complexes alone are susceptible to adsorption by serum proteins and degradation by nucleases, with a circulatory half-life of less than 2 h. Moreover, the non-specific cellular uptake of PEI causes siRNA release in non-target tissues, significantly reducing therapeutic efficacy and increasing side effects. PLGA, a biodegradable polymer approved by the FDA and EMA, has a hydrophobic backbone that partially neutralizes PEI ‘s positive charge, reducing non-specific interactions with cells [59]. Additionally, PLGA degradation yields lactic acid and glycolic acid, which are metabolized via the tricarboxylic acid cycle without risk of accumulation. The spherical structure of PLGA nanoparticles further enhances in vivo stability and controlled release of siRNA, protecting PEI-siRNA complexes from nuclease degradation [60].

Our experimental results confirmed that the M2M@NP complexes exhibited a more uniform and stable particle size while retaining the key surface markers of M2 macrophage membranes (M2M). We evaluated the targeting ability, therapeutic efficacy, and biosafety of this system in I/R and UUO mouse models. Short-term assessments (up to 28 days), including organ morphology (H&E staining) and blood biochemical indicators, verified the biocompatibility of the M2M@NP delivery system. However, the long-term toxic risks of nanocarriers require systematic evaluation, integrating material properties, in vivo metabolic profiles, and evidence from analogous studies. The long-term safety of nanomaterials is primarily influenced by tissue accumulation, oxidative stress, immune activation, and off-target effects—risks that may not be apparent in short-term experiments. For the PLGA nanocarriers used in this study, Chiu et al. ‘s systematic review on the cytotoxicity of targeted PLGA nanoparticles indicated a negative correlation between particle size and toxicity. Although most PLGA nanoparticles smaller than 300 nm showed low toxicity in short-term studies, long-term exposure may cause potential damage due to slow accumulation in metabolic organs such as the liver and spleen. Particularly, nanoparticles smaller than 10 nm are more likely to penetrate tissue barriers and form persistent deposits. This observation aligns with the small peak at approximately 10 nm in the particle size distribution observed in our study, suggesting the need to monitor the long-term accumulation risk of this particle subpopulation [61, 62]. For siRNA delivery systems, the cumulative risk of off-target effects also warrants careful consideration in the context of long-term toxicity. Off-target effects primarily arise from mismatched binding of the RNA-induced silencing complex (RISC) to non-target mRNAs, or from non-specific inhibition of unintended genes by the sense strand. These effects may be undetectable during short-term administration due to low siRNA concentrations; however, long-term sustained gene silencing pressure could disrupt metabolic pathways or impair normal cellular functions. Addressing these potential risks will be a key focus in our future experimental investigations [63, 64].

Renal fibrosis represents a common pathway in the progression of CKD, yet effective anti-fibrotic therapies capable of truly reversing fibrosis remain lacking in clinical practice. Current strategies primarily aim to modulate fibrotic signaling pathways, such as TGF-β and RAAS, or to suppress inflammatory responses; however, they are limited by modest efficacy, poor targeting, and systemic side effects. The M2M@NP-ADAM9 siRNA system developed in this study offers distinct advantages over existing approaches in terms of mechanism of action, targeting specificity, and therapeutic strategy, although it also presents its own set of challenges.

The M2M@NP-ADAM9 siRNA system regulates the AKT/NF-κB-mediated inflammatory pathway and fibrotic processes, thereby silencing the pathogenic transcriptional programs that drive this fibrotic response. The M2 macrophage membrane confers inflammatory chemotaxis and homotypic targeting capabilities, directing the nanocarriers toward inflammatory endothelial cells and renal parenchymal cells and significantly enhancing renal accumulation. In addition, the biologically derived membrane coating offers excellent biocompatibility and low immunogenicity. Importantly, siRNA-based therapy is sequence-specific and reversible, with off-target effects that can be rationally controlled.

In contrast, existing anti-fibrotic drugs, such as pirfenidone, exhibit broad pharmacological effects but relatively low target specificity [49, 50]. Most of these agents are systemically distributed with limited tissue targeting, necessitating higher doses to achieve therapeutic concentrations at lesion sites. This lack of specificity is a major contributor to systemic adverse effects, including hypotension, hepatotoxicity, and gastrointestinal reactions.

To facilitate the clinical translation of M2M@NP-ADAM9 siRNA, our future studies will focus on direct comparisons with clinically approved anti-fibrotic drugs, evaluating whether M2M@NP-20 μg ADAM9 siRNA can achieve superior therapeutic efficacy with improved safety profiles.

The scalability of macrophage isolation and preparation technologies represents a critical challenge for clinical translation and large-scale applications. Current mainstream strategies include magnetic-activated cell sorting (MACS) and fluorescence-activated cell sorting (FACS) based on specific surface markers (e.g., CD14 and CD11b), as well as in vitro polarization induction approaches (e.g., IL-4/IL-13-induced M2 phenotype). Although these approaches are well established at the laboratory scale, their industrial-level implementation remains constrained by high costs, stringent process requirements, and challenges in preserving cell viability, functional integrity, and phenotypic stability during large-scale production. Further research remains imperative to address these limitations.

## Conclusion

In this study, we employed a novel approach using M2 macrophage membrane-coated PEI/PLGA nanoparticles (referred to as M2M@NP complexes) for the targeted delivery of drugs to treat I/R- or UUO-induced kidney injury. The M2M@NP delivery system demonstrated several unique advantages, allowing precise targeting to the damaged renal area while ensuring safety to regular tissues and extending fast-acting anti-inflammatory effects to renal tubular cells. During the IR treatment process, M2M@NP-ADAM9 siRNA significantly suppressed P-AKT and NF-κB levels, which attenuated the inflammatory response, improved renal function, and ameliorated renal fibrosis during the recovery phase. These findings indicate a close association between ADAM9 and triggering of the AKT/NF-κB signaling pathway. Importantly, the M2M@NP complex retained its macrophage-targeting ability in in vivo experiments, enabling it to specifically locate injured or inflamed areas. Our findings suggest that the M2M@NP delivery system has potential as a novel therapeutic approach for treating kidney injuries.

## Data Availability

No datasets were generated or analysed during the current study.

## Abbreviations

ADAM9: A disintegrin and metalloproteinase 9
NF-κB: Nuclear factor kappa-light-chain-enhancer of activated B cells
P-Akt: Phosphorylated protein kinase B
α-SMA: Alpha-smooth muscle actin
Col-I: Collagen type I
IL-6: Interleukin 6
TNF-α: Tumor necrosis factor alpha
CD206: Cluster of differentiation 206
CD14: Cluster of differentiation 14
Rac1: Ras-related C3 botulinum toxin substrate 1
TRAF6: TNF receptor associated factor 6
GAPDH: Glyceraldehyde 3-phosphate dehydrogenase
GO: Gene ontology
KEGG: Kyoto encyclopedia of genes and genomes
FBS: Fetal bovine serum
PBS: Phosphate-buffered saline
PCR: Polymerase chain reaction
H&E: Hematoxylin and eosin
DAPI: 4 ‘,6-Diamidino-2-phenylindole
FDR: False discovery rate
